# New hydrated phases of potassium orthovanadate: K_3_(VO_4_)(H_2_O)_0.56_ and K_3_(VO_4_)(H_2_O)_4_

**DOI:** 10.1107/S2056989025008722

**Published:** 2025-10-09

**Authors:** Tobias Wolflehner, Matthias Weil

**Affiliations:** a Institute for Chemical Technologies and Analytics, Division of Applied Solid State Chemistry, Getreidemarkt 9/E164-05-1, 1060 Vienna, Austria; University of Aberdeen, United Kingdom

**Keywords:** crystal structure, orthovanadate, hydrogen bonding, hydrate, hydro­flux synthesis

## Abstract

Single crystals of the hydrated potassium orthovanadate phases K_3_(VO_4_)·0.56H_2_O, tripotassium orthovanadate 0.56-hydrate, and K_3_(VO_4_)·4H_2_O, tripotassium orthovanadate tetra­hydrate, were isolated during the processing of products obtained under hydro­flux conditions. The asymmetric unit of K_3_(VO_4_)(H_2_O)_0.56_ (space group *P*1, *Z* = 12) comprises six formula units of K_3_(VO_4_) and five water mol­ecules of crystallization. The complex crystal structure is made up of isolated [VO_4_]^3–^ tetra­hedra connected *via* K^+^ cations exhibiting coordination numbers between 6 and 8. The structure is consolidated by the formation of a finite hydrogen-bonded network between the water mol­ecules and [VO_4_]^3–^ tetra­hedra. Two of the K^+^ cations are positionally disordered, and three of the water mol­ecules of crystallization are occupationally and/or positionally disordered. The asymmetric unit of K_3_(VO_4_)(H_2_O)_4_ (space group *Pmn*2_1_, *Z* = 2) comprises two K^+^ cations, one V^V^ atom, three O atoms and two water mol­ecules. The crystal structure is also made up from isolated [VO_4_]^3–^ tetra­hedra inter­linked by K^+^ cations (coordination numbers 7 and 8). In comparison with the less-hydrated phase, the higher water content of this compound results in an infinite network of O—H⋯O hydrogen bonds between the water mol­ecules and [VO_4_]^3–^ tetra­hedra.

## Abstract 2

The crystal structures of the two title hydrated phases of K_3_(VO_4_) consist of isolated vanadate tetra­hedra linked *via* K^+^ cations. The difference in water content is noticeable in the hydrogen-bonding inter­actions, which define a finite network for the 0.56-hydrate phase and an infinite network for the 4-hydrate phase.

## 1. Chemical context

Research into layered oxides with honeycomb structures is continuing due to the fascinating electronic, magnetic, quantum, and chemical properties of these functional mat­erials (Kanyolo *et al.*, 2023 ▸). Formation studies (Wolflehner, 2025 ▸) on new honeycomb oxido­anti­monates(V) with general formula *A*^I^_3*x*–1_(*M*^II^_*x*_Sb^V^_1–*x*_)O_2_ (where *A*^I^ denotes an alkali metal and *M*^II^ a first-row transition metal) unexpectedly led to the discovery of new phases in the *A*^I^_2_*X*^III^_2_Sb^III^_2_O_7_ system (*X*^III^ = Al, Fe, Ga), for which K_2_Al_2_Sb_2_O_7_ (Hirschle & Röhr, 2000 ▸), Rb_2_Al_2_Sb_2_O_7_, Cs_2_Al_2_Sb_2_O_7_ and the isotypic Cs_2_Al_2_As_2_O_7_ (Emmerling *et al.*, 2005 ▸) are known. During these studies, various homo- and heterovalent substitutions of *A*^I^, *X*^III^ and Sb^III^ were tested, including the use of vanadium as *X*^III^ under hydro­flux conditions. Background and details of this rather new synthesis method were recently compiled by He *et al.* (2023 ▸).

Under the given hydro­flux conditions, a redox reaction took place between parts of employed Sb^III^- and V^III^-containing precursors, which yielded elemental anti­mony and V^V^-containing orthovanadate. Single crystals of the hydrated potassium orthovanadate phases K_3_(VO_4_)(H_2_O)_0.56_ and K_3_(VO_4_)(H_2_O)_4_ were isolated during the processing of the products obtained from the hydro­flux experiment.

## 2. Structural commentary

### 2.1. K_3_(VO_4_)(H_2_O)_0.56_

The asymmetric unit of triclinic K_3_(VO_4_)(H_2_O)_0.56_ comprises six formula units of K_3_(VO_4_) and five water mol­ecules of crystallization, three of which (O2*W*, O3*W*, O5*W*) are disordered around centres of inversion. Assuming the highest possible occupancy (0.5) for disordered O3*W* [refined occupancy 0.364 (2)] would result in a composition of K_3_(VO_4_)(H_2_O)_0.583._

**Figure 1:**
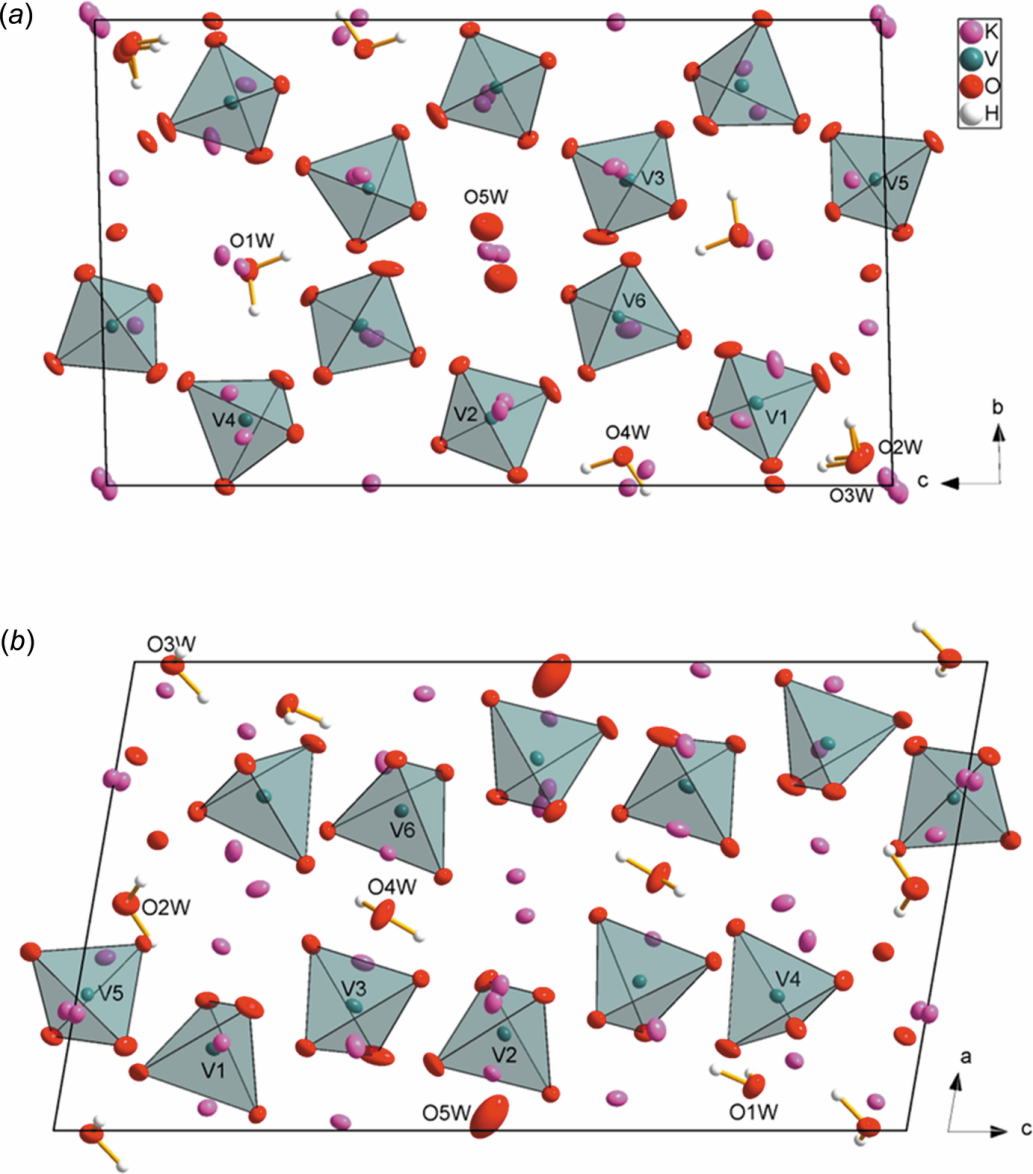
Crystal structure of K_3_(VO_4_)(H_2_O)_0.56_ in projections along [100] (*a*) and [010] (*b*). Displacement ellipsoids are given at the 75% probability level except for H atoms, which are shown with an arbitrary radius.

**Figure 2:**
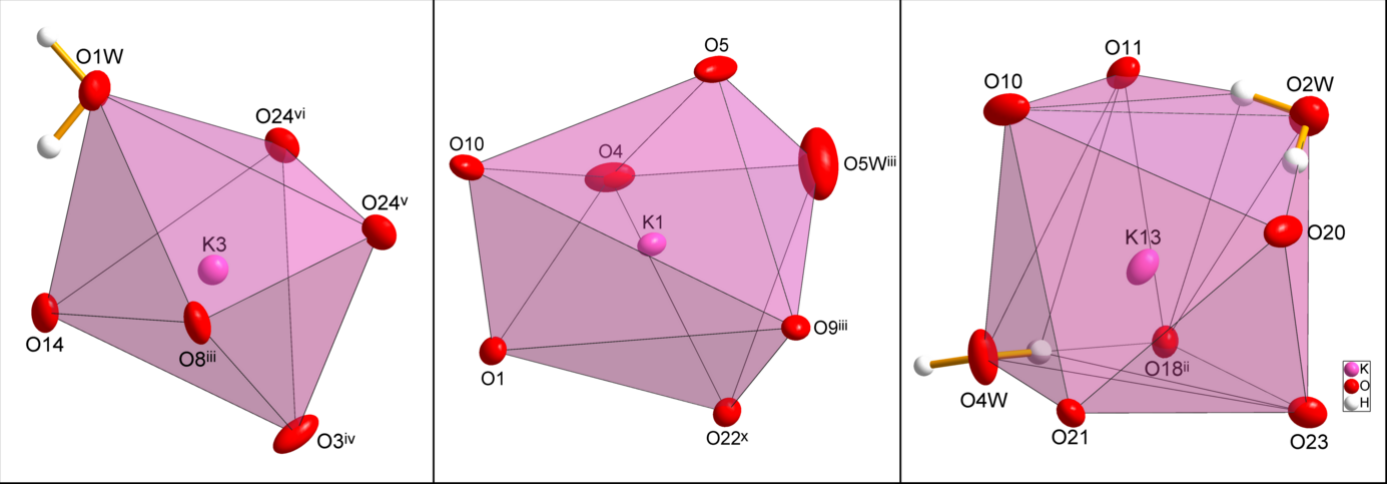
Representative coordination polyhedra for three K^+^ sites (K3, K1 and K13) in the crystal structure of K_3_(VO_4_)(H_2_O)_0.56_ with coordination numbers of 6, 7 and 8, respectively. Displacement ellipsoids are as in Fig. 1 ▸. [Symmetry codes: (ii) −*x* + 1, −*y*, −*z* + 1; (iii) −*x*, −*y* + 1, −*z* + 1; (iv) *x*, *y*, *z* + 1; (v) *x* − 1, *y*, *z* + 1; (vi) −*x* + 1, −*y* + 1, −*z* + 1; (*x*) *x* − 1, *y*, *z*.]

The crystal structure (Fig. 1 ▸) consists of isolated [VO_4_]^3–^ tetra­hedra, which are surrounded by K^+^ cations and water mol­ecules. The V—O distances in the six orthovanadate groups show a narrow range between 1.7062 (9) and 1.7379 (10) Å with an average of 1.718 (8) Å, in nearly perfect agreement with the literature value of 1.717 (56) Å (Gagné & Hawthorne, 2020 ▸). The slight angular distortions of the [VO_4_]^3–^ tetra­hedra are seen by the variation of the O—V—O angles ranging from 106.66 (4) to 111.43 (5)° with an average of 109.5(1.3)°, which is very close to the ideal tetra­hedral angle of 109.47°. Two (K7, K8) of the 18 K^+^ sites are disordered over two positions. The ordered K^+^ sites show coordination numbers between 6 and 8; representative polyhedra for the three coordination numbers are shown in Fig. 2 ▸. The description of the closest matching ideal polyhedron and qu­anti­fication of the distortion (*δ*) from it was performed for each ordered K^+^ site with the *Polynator* program (Link & Niewa, 2023 ▸). Idealized polyhedra and numerical data considering K—O distances up to 3.2 Å as relevant are compiled in Table 1 ▸, including averaged K—O bond lengths. The latter are in reasonable agreement with literature values (Gagné & Hawthorne, 2016 ▸) of 2.828 (177) Å for coordination number 6, 2.861 (179) Å for coordination number 7 and 2.894 (172) Å for coordination number 8.

**Figure 3:**
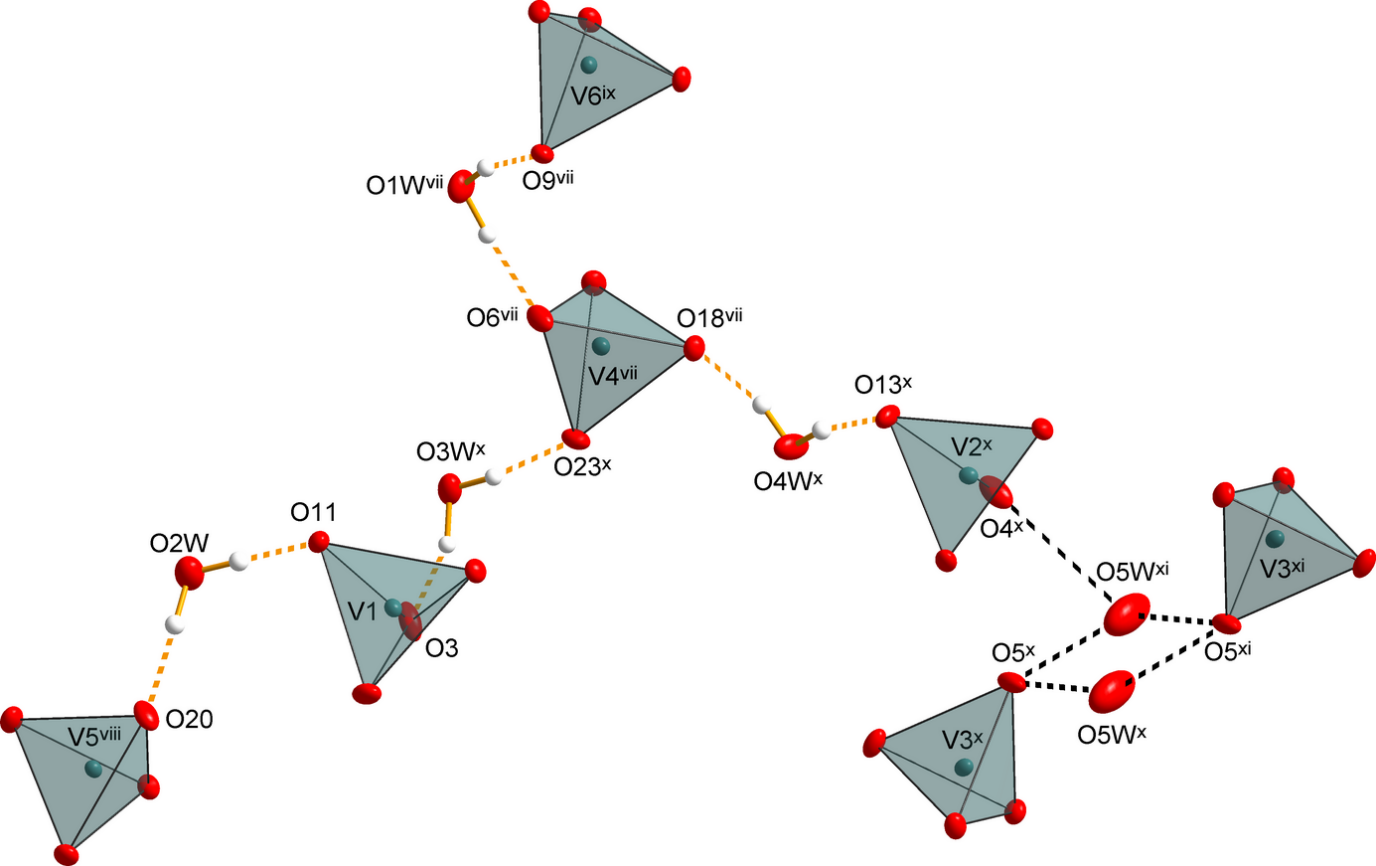
Finite hydrogen-bonding network (yellow dashed lines) in K_3_(VO_4_)(H_2_O)_0.56;_ possible hydrogen bonds with O*W*5 as donor group are given as black dashed lines. Note that positional disorder of water mol­ecules O2*W* and O3*W* is not shown. Displacement ellipsoids are as in Fig. 1 ▸. [Symmetry codes: (vii) −*x*, −*y*, −*z* + 1; (viii) −*x* + 1, −*y* + 1, −*z*; (ix) *x* − 1, *y* − 1, *z*; (*x*) *x* − 1, *y*, *z*; (xi) −*x* − 1, −*y* + 1, −*z* + 1.]

The crystal structure of K_3_(VO_4_)(H_2_O)_0.56_ is consolidated by O—H⋯O hydrogen bonds between water mol­ecules as donor groups and vanadate O atoms as acceptor atoms (Table 2 ▸). Based on corresponding *D*⋯*A* separations, the hydrogen-bonding inter­actions are considered to be of medium strength (Jeffrey, 1997 ▸). All water mol­ecules for which the H positions could be localized (O1*W*–O4*W*) are part of these inter­actions, bridging neighbouring vanadate tetra­hedra (centered by V1, V2, V4, V5 and V6) into a finite hydrogen-bonded network. However, the water mol­ecule O*W*5, for which no H-atom positions could be localized, also appears to be involved in the formation of this finite network, with O4 and O5 being the only meaningful acceptor O atoms, so that the vanadate tetra­hedron formed by V3 is also included in the network formation (Table 2 ▸, Fig. 3 ▸).

### 2.2. K_3_(VO_4_)(H_2_O)_4_

K_3_(VO_4_)(H_2_O)_4_ crystallizes in the non-centrosymmetric space group *Pmn*2_1_ and comprises two K^+^ cations, one V^V^ atom, three O atoms and two water mol­ecules in the asymmetric unit, with one K^+^ cation (K2), the V^V^ atom (V1) and two O atoms (O1, O2) located on a mirror plane.

**Figure 4:**
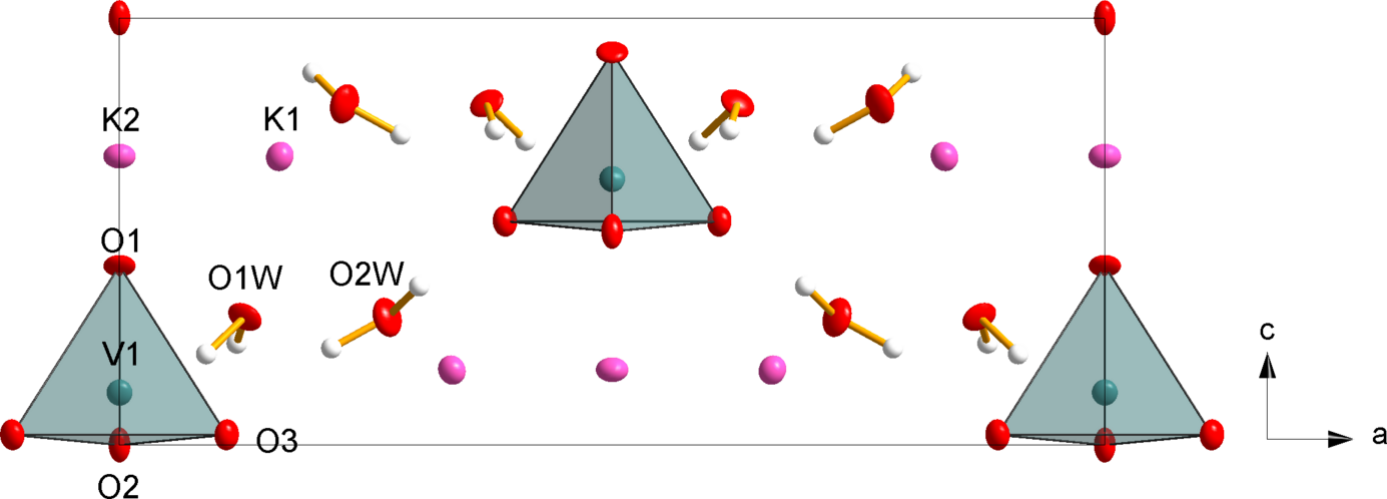
Crystal structure of K_3_(VO_4_)(H_2_O)_4_ in a projection along [010]. Displacement ellipsoids are as in Fig. 1 ▸.

As with the less-hydrated phase, the crystal structure of K_3_(VO_4_)(H_2_O)_4_ (Fig. 4 ▸) consists of isolated [VO_4_]^3–^ tetra­hedra, which are surrounded by K^+^ cations and water mol­ecules. The isolated vanadate tetra­hedron exhibits V—O bond lengths in the range of 1.667 (4)–1.736 (5) Å, with slight angular distortions from the ideal value, being in the range 108.45 (14)–110.6 (2)°. The two K^+^ cations have coordination numbers of 7 (K1) and 8 (K2) with similar K—O distances and distorted coordination polyhedra as in K_3_(VO_4_)(H_2_O)_0.56_. Due to the higher water content of K_3_(VO_4_)(H_2_O)_4_, both K^+^ cations show an increased number of coordinating water atoms compared to the less hydrated one, namely four for both cations (Table 1 ▸).

**Figure 5:**
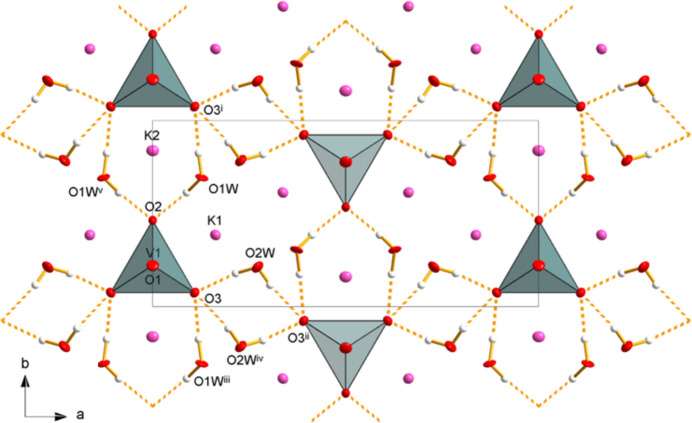
Hydrogen-bonding network (yellow dashed lines) in K_3_(VO_4_)(H_2_O)_4_ as seen in a projection along [001]. Displacement ellipsoids are as in Fig. 4 ▸. [Symmetry codes: (i) *x*, *y* + 1, *z*; (ii) −*x* + 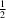, −*y*, *z* + 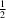; (iii) *x*, *y* − 1, *z*; (iv) −*x* + 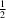, −*y*, *z* − 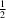; (v) −*x*, *y*, *z*.]

The higher water content of the tetra­hydrate phase also defines a different hydrogen-bonding scheme compared to the less-hydrated phase. Here, the O—H⋯O inter­actions (Table 3 ▸) are not limited to a finite hydrogen-bonding network but form a tri-periodic arrangement (Fig. 5 ▸). Again, these hydrogen bonds can be rationalized as inter­actions between the two water mol­ecules and the [VO_4_]^3–^ tetra­hedron. Thereby, O2 is a double acceptor atom (in addition to the entry in Table 3 ▸ there is another O1*W* donor group symmetry-related through the mirror plane) whereas O3 acts as a triple acceptor of the overall medium–strong inter­actions. The acceptor properties of O2 and O3 also have an effect on the V—O bond lengths, which are significantly longer for O2 [1.736 (5) Å] and O3 [2 × 1.732 (3) Å] than for O1 [1.667 (4) Å], which is not involved in the formation of the hydrogen-bonding network.

### 2.3. Bond-valence-sum calculation

For both K_3_(VO_4_) hydrates, calculations of bond valence sums (BVS; Brown, 2002 ▸) were performed with the program *ECoN21* (Ilinca, 2022 ▸) to verify the plausibility of the structure models. The BVS values of all atomic sites are listed in Table 4 ▸ and correspond to expectations [1.00 valence unit (v. u.) for K, 5.00 v. u. for V, 2.00 v. u. for O]. They also reflect the role of individual oxygen atoms in hydrogen-bonding inter­actions. Since the contributions of H atoms to the bonding was not taken into account in the calculations, the O atoms of the water mol­ecules (O*W) have very low BVS values, and the O atoms acting as an acceptor of a hydrogen bond (Tables 2 ▸, 3 ▸) show a value significantly below 2. For K_3_(VO_4_)(H_2_O)_0.56_ this also includes O4 and O5 as potential acceptor atoms of O5*W*, which can be seen as a further argument for the plausibility of this undetermined hydrogen bond.

## 3. Database survey

A search of the Inorganic Crystal Structure Database (ICSD; data release 2024-1; Zagorac *et al.*, 2019 ▸) for K_3_(*X*O_4_)(H_2_O)_*n*_ phases with tetra­hedral anions (*X* = P, As, V) revealed one entry for orthophosphates, K_3_(PO_4_)(H_2_O)_7_ (Weil & Stöger, 2020 ▸), no entry for orthoarsenates, and one entry for orthovanadates, K_3_(VO_4_)(H_2_O) (Kato & Takayama-Muromachi, 1987 ▸). While potassium orthophosphate hepta­hydrate has a different structural arrangement compared to the title compounds due to its higher water content, potassium orthovanadate monohydrate shows a certain degree of similarity to K_3_(VO_4_)(H_2_O)_0.56_. K_3_(VO_4_)(H_2_O) crystallizes with one formula unit in the asymmetric unit and has an ortho­rhom­bic crystal structure (space group *Pbca*; *Z* = 8) with *a* = 10.2136 (8), *b* = 10.4447 (8), *c* = 12.4878 (6) Å and a cell volume of 1332.17 (16) Å^3^ at room temperature. A comparison of the two unit cells shows that the *a* and *b* axes are similar and the *c* axis of K_3_(VO_4_)(H_2_O)_0.56_ is elongated by a factor of about 1.5 relative to K_3_(VO_4_)(H_2_O).

## 4. Synthesis and crystallization

Single crystals of K_3_(VO_4_)(H_2_O)_0.56_ and K_3_(VO_4_)(H_2_O)_4_ were isolated during the processing of products obtained under hydro­flux conditions. In a first batch, V_2_O_3_ and Sb_2_O_3_ powders and ground KOH pellets (85%_wt_) were mixed in a 1:1:12 molar ratio and put into a Teflon container without addition of water. The container was closed with a teflon lid and placed in a steel autoclave, which was heated at 483 K for 18 h and then cooled to room temperature within three h. Under the given conditions, parts of employed Sb^III^ and V^III^ underwent a redox reaction, producing elemental Sb and the orthovanadate anion, (V^V^O_4_)^3–^. The obtained grey product was washed twice with dry methanol and dry iso­propanol to leach out excess KOH flux, followed by drying under vacuum for 10 min at room temperature. Crystals of K_3_(VO_4_)(H_2_O)_0.56_ were manually separated from the final product. In a second batch serving to reproduce synthesis of this phase, a molar 1:25:3 mixture of V_2_O_5_, KOH (85%_wt_) and deionized water was used under otherwise identical conditions. From this batch, single crystals of K_3_(VO_4_)(H_2_O)_4_ were isolated from the final off-white product. Powder X-ray diffraction revealed elemental Sb and K_3_(VO_4_)(H_2_O)_4_ as the main product phases present in the first batch, and K_3_(VO_4_)(H_2_O)_4_ as the main phase in the second batch.

## 5. Refinement

**Figure 6:**
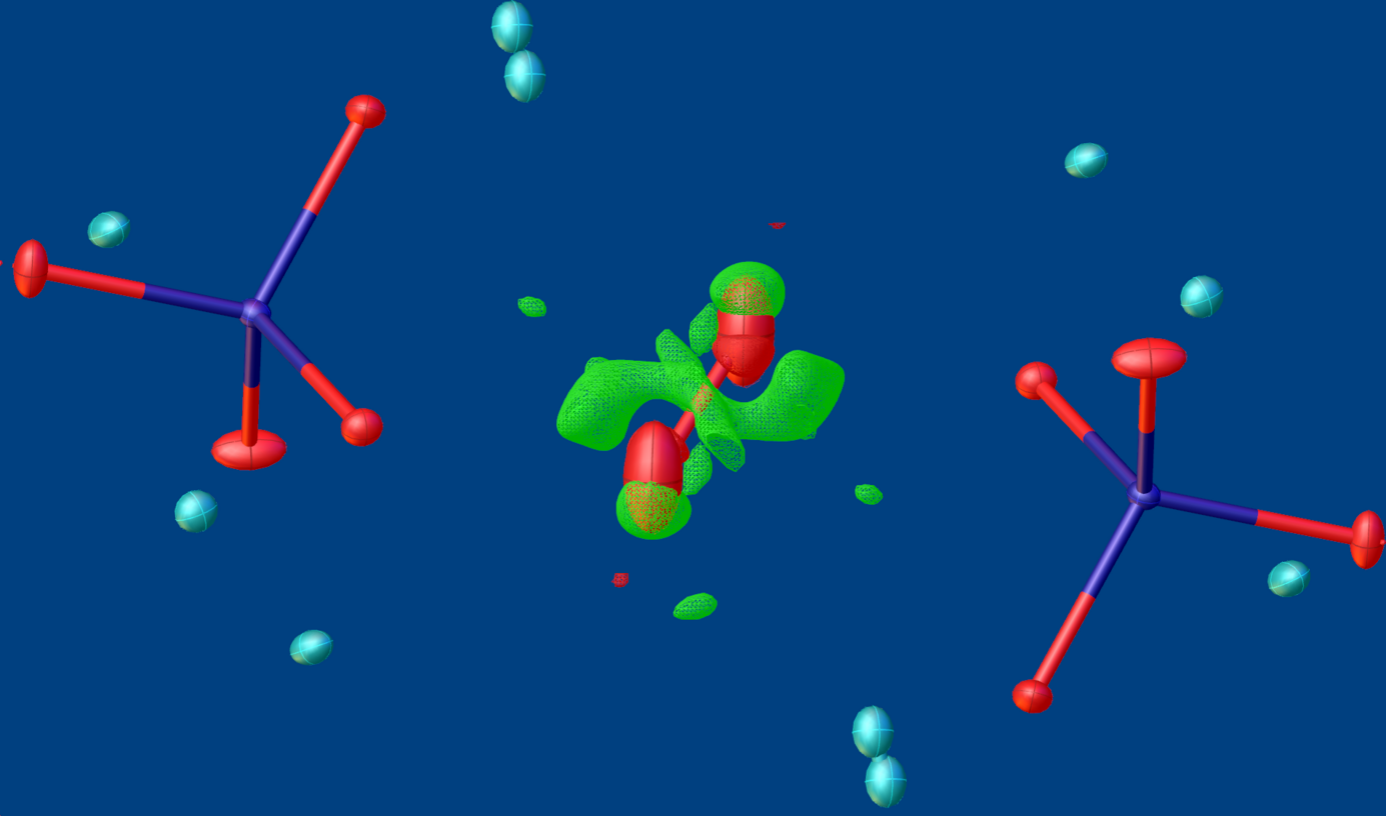
Difference-Fourier map centered on O5*W*, showcasing the highly diffuse remaining electron density. The wire style map was created by *OLEX2*, with electron density ranges from −1.05 to +1.50 *e* Å^−3^.

Crystal data, data collection and structure refinement details are summarized in Table 5 ▸. Structure data of both compounds were standardized with *STRUCTURE TIDY* (Gelato & Parthé, 1987 ▸). In the K_3_(VO_4_)(H_2_O)_0.56_ structure, three (O2*W*, O3*W* and O5*W*) of the five water mol­ecules of crystallization are disordered about inversion centres and thus show positional disorder with maximal possible occupancy of 0.5 for O2*W* and O5*W*, and a refined occupancy of 0.364 (2) for O3*W*. Two K^+^ sites to which the disordered water mol­ecules are bound are likewise positionally disordered, *viz*. K7 and K8, with ratios of K7*A*:K7*B* = 0.563 (3):0.437 (3) and of K8*A*:K8*B* = 0.876 (6):0.124 (6). Hydrogen positions of water mol­ecules O1*W*–O4*W* were clearly discernible from difference-Fourier maps; the O—H bond lengths were restrained to 0.96 (1) Å with *U*_iso_(H) = 1.5*U*_eq_(O). Reasonable H atom positions could not be derived for O5*W*. As shown in Fig. 6 ▸, the electron density is extremely smeared, which hints at further disorder than considered in the final model. Attempts to further split the O5*W* position and locate the hydrogen atoms failed. Hence, the corresponding H atoms are not part of the crystal structure model but are considered in the crystallographic data.

For K_3_(VO_4_)(H_2_O)_4_ all sites show full occupancy; the water H atoms were located from difference-Fourier maps and were refined with the same restraints as noted above.

## Supplementary Material

Crystal structure: contains datablock(s) K3VO4_0.56H2O, K3VO4_4H2O, global. DOI: 10.1107/S2056989025008722/hb8164sup1.cif

Structure factors: contains datablock(s) K3VO4_0.56H2O. DOI: 10.1107/S2056989025008722/hb8164K3VO4_0.56H2Osup2.hkl

Structure factors: contains datablock(s) K3VO4_4H2O. DOI: 10.1107/S2056989025008722/hb8164K3VO4_4H2Osup3.hkl

CCDC references: 2493515, 2493514

Additional supporting information:  crystallographic information; 3D view; checkCIF report

## Figures and Tables

**Table 1:** Coordination environments around the ordered K^+^ cations in K_3_(VO_4_)(H_2_O)_0.56_ and K_3_(VO_4_)(H_2_O)_4_

Atom	Coordination number	Polyhedron with idealized point group symmetry [in brackets] and deviation *δ* (in parentheses) from it	Range of K—O bond lengths (Å)	Average K—O bond length (Å)	Number of water mol­ecules in the first coordination sphere (< 3.2 Å)
**K_3_(VO_4_)(H_2_O)_0.56_**					
K1	7	monocapped isosceles wedge [*mm*2] (22.703)	2.6147 (10)–3.1147 (11)	2.820	1; O5*W*
K2	7	monocapped trigonal anti­frustum [3*m*] (17.810)	2.5976 (10)–2.9519 (10)	2.780	1; O3*W*
K3	6	twisted trigonal prism [32] (14.537)	2.6578 (9)–2.8978 (10)	2.809	1; O1*W*
K4	6	twisted trigonal prism [32] (20.715)	2.6509 (9)–2.8147 (9)	2.727	–
K5	7	penta­gonal heterobipyramid [5*m*] (16.606)	2.5843 (9)–3.159 (3)	2.814	1; O5*W*
K6	6	isosceles wedge [*mm*2] (11.584)	2.6495 (10)–3.0826 (11)	2.876	1; O1*W*
K9	7	penta­gonal heterobipyramid [5*m*] (14.671)	2.6385 (11)–3.0139 (11)	2.846	1; O4*W*
K10	7	monocapped isosceles wedge [*mm*2] (18.963)	2.7573 (9)–3.087 (2)	2.841	1; O2*W*
K11	7	monocapped isosceles wedge [*mm*2] (20.596)	2.6906 (10)–2.9803 (10)	2.830	–
K12	7	monocapped isosceles wedge [*mm*2] (19.983)	2.7139 (9)–3.0687 (10)	2.829	–
K13	8	biaugmented isosceles wedge [*mm*2] (16.732)	2.6615 (9)–2.9768 (10)	2.833	2; O4*W*, O2*W*
K14	6	twisted trigonal prism [32] (21.107)	2.5959 (10)–2.9085 (10)	2.759	–
K15	6	bailar twist (dynamic) [32] (20.067)	2.7112 (10)–3.0799 (11)	2.902	–
K16	6	twisted trigonal prism [32] (20.309)	2.5582 (10)–3.1992 (10)	2.943	–
K17	6	trigonal anti­frustum [3*m*] (18.065)	2.6822 (10)–3.1162 (11)	2.899	1; O4*W*
K18	8	biaugmented isosceles wedge [*mm*2] (11.911)	2.7547 (9)–3.1367 (12)	2.931	1; O1*W*
					
**K_3_(VO_4_)(H_2_O)_4_**					
K1	7	monocapped isosceles wedge [*mm*2] (9.434)	2.735 (4)–2.894 (3)	2.809	4; O2*W*, O2*W*′, O1*W*, O1*W*′
K2	8	hexa­gonal heterobipyramid [6*mm*2] (15.223)	2.798 (5)–3.056 (3)	2.897	4; O1*W*, O1*W*′, O2*W*, O2*W*′

**Table 2:** Hydrogen-bond geometry (Å, °) for K_3_(VO_4_)(H_2_O)_0.56_

*D*—H⋯*A*	*D*—H	H⋯*A*	*D*⋯*A*	*D*—H⋯*A*
O1*W*—H1*A*⋯O6	0.94 (1)	1.72 (1)	2.6483 (15)	167 (2)
O1*W*—H1*B*⋯O9	0.94 (1)	1.68 (1)	2.6057 (14)	167 (2)
O2*W*—H2*A*⋯O11	0.97 (1)	1.72 (1)	2.676 (2)	171 (4)
O2*W*—H2*B*⋯O20	0.96 (1)	1.67 (1)	2.630 (2)	173 (4)
O3*W*—H3*A*⋯O23	0.96 (1)	1.70 (2)	2.641 (3)	166 (5)
O3*W*—H3*B*⋯O3^i^	0.96 (1)	1.70 (3)	2.559 (3)	148 (5)
O4*W*—H4*B*⋯O13	0.93 (1)	1.71 (1)	2.6303 (14)	168 (2)
O4*W*—H4*A*⋯O18^ii^	0.94 (1)	1.76 (1)	2.6710 (14)	162 (2)
O5*W*⋯O5			2.725 (3)	
O5*W*⋯O4^iii^			3.089 (3)	

Symmetry codes: (i) 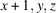; (ii) 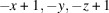; (iii) 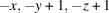.

**Table 3:** Hydrogen-bond geometry (Å, °) for K_3_(VO_4_)(H_2_O)_4_

*D*—H⋯*A*	*D*—H	H⋯*A*	*D*⋯*A*	*D*—H⋯*A*
O1*W*—H1*A*⋯O2	0.87 (3)	1.90 (3)	2.759 (5)	174 (4)
O1*W*—H1*B*⋯O3^i^	0.90 (3)	1.98 (3)	2.855 (5)	165 (3)
O2*W*—H2*A*⋯O3	0.86 (3)	1.90 (3)	2.753 (4)	168 (4)
O2*W*—H2*B*⋯O3^ii^	0.84 (4)	1.86 (4)	2.696 (4)	172 (4)

Symmetry codes: (i) 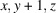; (ii) 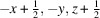.

**Table 4:** BVS calculations for K_3_(VO_4_)(H_2_O)_0.56_ and K_3_(VO_4_)(H_2_O)_4_ without contributions of H atoms

Atom	Occupancy	BVS (valence units)
**K_3_(VO_4_)(H_2_O)_0.56_**		
K1	1	1.08
K2	1	1.11
K3	1	0.94
K4	1	1.16
K5	1	1.08
K6	1	0.90
K7*A*	0.563 (3)	0.57
K7*B*	0.437 (3)	0.46
K8*A*	0.876 (6)	0.82
K8*B*	0.124 (6)	0.12
K9	1	1.01
K10	1	1.01
K11	1	1.03
K12	1	1.03
K13	1	1.10
K14	1	1.10
K15	1	0.78
K16	1	0.85
K17	1	0.84
K18	1	0.88
V1	1	4.93
V2	1	4.96
V3	1	4.91
V4	1	4.95
V5	1	4.96
V6	1	4.96
O1	1	1.92
O2	1	1.90
O3	1	1.82
O4	1	1.91
O5	1	1.81
O6	1	1.79
O7	1	2.01
O8	1	1.97
O9	1	1.82
O10	1	1.94
O11	1	1.87
O12	1	2.05
O13	1	1.83
O14	1	1.82
O15	1	1.89
O16	1	2.00
O17	1	2.02
O18	1	1.81
O19	1	2.01
O20	1	1.83
O21	1	1.90
O22	1	2.16
O23	1	1.86
O24	1	1.93
O1*W*	1	0.46
O2*W*	0.5	0.32
O3*W*	0.350 (4)	0.23
O4*W*	1	0.47
O5*W*	0.5	0.21
		
**K_3_(VO_4_)(H_2_O)_4_**		
K1	1	1.06
K2	1	0.99
V1	1	5.01
O1	1	1.90
O2	1	1.55
O3	1	1.47
O1*W*	1	0.42
O2*W*	1	0.45

**Table 5:** Experimental details

	K_3_(VO_4_)·0.56H_2_O	K_3_(VO_4_)·4H_2_O
Crystal data		
*M*_r_	242.33	304.30
Crystal system, space group	Triclinic, *P* 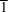	Orthorhombic, *Pmn*2_1_
Temperature (K)	100	100
*a*, *b*, *c* (Å)	10.0469 (4), 10.5183 (4), 18.0052 (7)	12.9607 (13), 6.2512 (6), 5.6130 (8)
α, β, γ (°)	88.069 (3), 80.105 (3), 87.310 (3)	90, 90, 90
*V* (Å^3^)	1871.72 (13)	454.76 (9)
*Z*	12	2
Radiation type	Mo *K*α	Mo *K*α
μ (mm^−1^)	3.53	2.47
Crystal size (mm)	0.10 × 0.08 × 0.06	0.10 × 0.04 × 0.03

Data collection		
Diffractometer	Stoe STADIVARI	Stoe STADIVARI
Absorption correction	Multi-scan (*LANA*; Koziskova *et al.*, 2016 ▸)	Multi-scan (*LANA*; Koziskova *et al.*, 2016 ▸)
*T*_min_, *T*_max_	0.483, 0.696	0.576, 0.895
No. of measured, independent and observed reflections	63248, 17892, 15189 [*I* > 2σ(*I*)]	6297, 1603, 1018 [*I* > 2s(*I*)]
*R*_int_	0.018	0.057
(sin θ/λ)_max_ (Å^−1^)	0.844	0.757

Refinement		
*R*[*F*^2^ > 2σ(*F*^2^)], *wR*(*F*^2^), *S*	0.022, 0.052, 1.05	0.031, 0.051, 0.80
No. of reflections	17892	1603
No. of parameters	512	74
No. of restraints	8	7
H-atom treatment	Only H-atom coordinates refined	Only H-atom coordinates refined
Δρ_max_, Δρ_min_ (e Å^−3^)	1.09, −0.81	0.45, −0.79
Absolute structure	–	Classical Flack method preferred over Parsons because s.u. lower
Absolute structure parameter	–	0.05 (5)

Computer programs: *X-AREA* (Stoe, 2024 ▸), *SHELXT* (Sheldrick, 2015*a* ▸), *SHELXL* (Sheldrick, 2015*b* ▸), *OLEX2* (Dolomanov *et al.*, 2009 ▸) and *publCIF* (Westrip, 2010 ▸).

## supplementary crystallographic information

### Tripotassium orthovanadate 0.56-hydrate (K3VO4_0.56H2O) Crystal data

**Table:**

K_3_(VO_4_)·0.56H_2_O	*Z* = 12
*M**_r_* = 242.33	*F*(000) = 1411
Triclinic, *P*1	*D*_x_ = 2.580 Mg m^−^^3^
*a* = 10.0469 (4) Å	Mo *K*α radiation, λ = 0.71073 Å
*b* = 10.5183 (4) Å	Cell parameters from 9324 reflections
*c* = 18.0052 (7) Å	θ = 1.9–37.1°
α = 88.069 (3)°	µ = 3.53 mm^−^^1^
β = 80.105 (3)°	*T* = 100 K
γ = 87.310 (3)°	Block, colorless
*V* = 1871.72 (13) Å^3^	0.10 × 0.08 × 0.06 mm

### Tripotassium orthovanadate 0.56-hydrate (K3VO4_0.56H2O) Data collection

**Table:**

Stoe STADIVARI CCD diffractometer	17892 independent reflections
Radiation source: Axo_Mo	15189 reflections with *I* > 2σ(*I*)
Graded multilayer mirror monochromator	*R*_int_ = 0.018
rotation method, ω scans	θ_max_ = 36.9°, θ_min_ = 1.9°
Absorption correction: multi-scan (*LANA*; Koziskova *et al.*, 2016)	*h* = −16→16
*T*_min_ = 0.483, *T*_max_ = 0.696	*k* = −17→17
63248 measured reflections	*l* = −16→30

### Tripotassium orthovanadate 0.56-hydrate (K3VO4_0.56H2O) Refinement

**Table:**

Refinement on *F*^2^	Hydrogen site location: difference Fourier map
Least-squares matrix: full	Only H-atom coordinates refined
*R*[*F*^2^ > 2σ(*F*^2^)] = 0.022	*w* = 1/[σ^2^(*F*_o_^2^) + (0.0225*P*)^2^ + 1.1555*P*] where *P* = (*F*_o_^2^ + 2*F*_c_^2^)/3
*wR*(*F*^2^) = 0.052	(Δ/σ)_max_ = 0.003
*S* = 1.05	Δρ_max_ = 1.09 e Å^−^^3^
17892 reflections	Δρ_min_ = −0.81 e Å^−^^3^
512 parameters	Extinction correction: SHELXL-2019/2 (Sheldrick, 2015b), Fc^*^=kFc[1+0.001xFc^2^λ^3^/sin(2θ)]^-1/4^
8 restraints	Extinction coefficient: 0.00067 (6)

### Tripotassium orthovanadate 0.56-hydrate (K3VO4_0.56H2O) Special details

**Table:**

Geometry. All esds (except the esd in the dihedral angle between two l.s. planes) are estimated using the full covariance matrix. The cell esds are taken into account individually in the estimation of esds in distances, angles and torsion angles; correlations between esds in cell parameters are only used when they are defined by crystal symmetry. An approximate (isotropic) treatment of cell esds is used for estimating esds involving l.s. planes.

### Tripotassium orthovanadate 0.56-hydrate (K3VO4_0.56H2O) Fractional atomic coordinates and isotropic or equivalent isotropic displacement parameters (Å^2^)

**Table:**

	*x*	*y*	*z*	*U*_iso_*/*U*_eq_	Occ. (<1)
K1	0.01783 (3)	0.33326 (2)	0.33489 (2)	0.00932 (4)	
K2	0.04852 (3)	0.89596 (2)	0.17576 (2)	0.00930 (4)	
K3	0.06118 (3)	0.34346 (2)	0.95852 (2)	0.00893 (4)	
K4	0.06837 (3)	0.31528 (2)	0.65399 (2)	0.00989 (4)	
K5	0.12150 (3)	0.83909 (3)	0.50250 (2)	0.01029 (4)	
K6	0.18672 (3)	0.53136 (3)	0.17919 (2)	0.00973 (4)	
K7A	0.24835 (13)	0.00957 (13)	0.00352 (7)	0.01115 (13)	0.563 (3)
K7B	0.74403 (18)	0.01681 (16)	0.00937 (9)	0.01115 (13)	0.437 (3)
K8A	0.30817 (15)	0.49818 (4)	0.49428 (3)	0.01130 (13)	0.876 (6)
K8B	0.2732 (8)	0.4969 (4)	0.4897 (2)	0.01130 (13)	0.124 (6)
K9	0.35781 (3)	0.33471 (3)	0.32875 (2)	0.01144 (4)	
K10	0.36917 (3)	0.33895 (2)	0.02560 (2)	0.00962 (4)	
K11	0.39265 (3)	0.80190 (2)	0.15916 (2)	0.00894 (4)	
K12	0.40854 (2)	0.32186 (2)	0.66400 (2)	0.00818 (4)	
K13	0.51514 (3)	0.13962 (2)	0.19047 (2)	0.01109 (4)	
K14	0.54237 (2)	0.18059 (2)	0.49050 (2)	0.00897 (4)	
K15	0.59620 (3)	0.50620 (3)	0.15454 (2)	0.01262 (5)	
K16	0.78518 (3)	0.03395 (3)	0.31345 (2)	0.01292 (5)	
K17	0.82048 (3)	0.00467 (3)	0.66315 (2)	0.01266 (5)	
K18	0.85116 (3)	0.25997 (3)	0.14544 (2)	0.01443 (5)	
V1	0.17418 (2)	0.17735 (2)	0.17086 (2)	0.00616 (3)	
V2	0.20618 (2)	0.14475 (2)	0.50860 (2)	0.00595 (3)	
V3	0.26565 (2)	0.65611 (2)	0.32724 (2)	0.00688 (3)	
V4	0.28688 (2)	0.14080 (2)	0.82116 (2)	0.00627 (3)	
V5	0.29031 (2)	0.65775 (2)	0.01222 (2)	0.00556 (3)	
V6	0.68189 (2)	0.36156 (2)	0.34316 (2)	0.00560 (3)	
O1	0.03406 (9)	0.13933 (9)	0.23589 (5)	0.00960 (15)	
O2	0.07814 (9)	0.08714 (8)	0.57553 (5)	0.00887 (14)	
O3	0.12407 (10)	0.23043 (11)	0.08805 (5)	0.01605 (19)	
O4	0.14053 (11)	0.20567 (11)	0.43246 (6)	0.01673 (19)	
O5	0.15995 (10)	0.53431 (10)	0.36297 (7)	0.0193 (2)	
O6	0.17472 (10)	0.22698 (9)	0.77403 (6)	0.01394 (17)	
O7	0.17609 (9)	0.76434 (9)	0.27866 (5)	0.01050 (15)	
O8	0.17947 (10)	0.75897 (9)	0.06733 (5)	0.01334 (17)	
O9	0.20855 (9)	0.51489 (9)	0.67413 (5)	0.01044 (15)	
O10	0.26217 (10)	0.29139 (9)	0.20476 (6)	0.01507 (18)	
O11	0.27710 (9)	0.04236 (9)	0.15371 (5)	0.01109 (16)	
O12	0.28840 (9)	0.25781 (9)	0.54736 (5)	0.01082 (15)	
O13	0.31874 (10)	0.02185 (9)	0.47711 (5)	0.01296 (17)	
O14	0.31936 (10)	0.22288 (9)	0.89597 (5)	0.01200 (16)	
O15	0.32375 (9)	0.72408 (9)	0.39995 (5)	0.01040 (15)	
O16	0.39678 (10)	0.59306 (9)	0.26442 (5)	0.01181 (16)	
O17	0.39817 (9)	0.58756 (9)	0.06739 (5)	0.00939 (15)	
O18	0.43298 (9)	0.11124 (9)	0.75845 (5)	0.01063 (15)	
O19	0.54149 (9)	0.41051 (9)	0.40539 (5)	0.00973 (15)	
O20	0.61897 (10)	0.26140 (10)	0.06323 (5)	0.01310 (17)	
O21	0.63935 (9)	0.31389 (9)	0.26044 (5)	0.01005 (15)	
O22	0.76371 (9)	0.24116 (8)	0.38580 (5)	0.00936 (15)	
O23	0.78257 (10)	0.00252 (9)	0.15021 (5)	0.01166 (16)	
O24	0.79881 (9)	0.45625 (9)	0.02093 (5)	0.01120 (16)	
O1W	0.09397 (11)	0.46272 (10)	0.81219 (6)	0.01643 (18)	
H1A	0.121 (2)	0.3760 (10)	0.8061 (12)	0.025*	
H1B	0.130 (2)	0.494 (2)	0.7640 (7)	0.025*	
O2W	0.4837 (2)	0.0632 (2)	0.03897 (13)	0.0198 (4)	0.5
H2A	0.403 (3)	0.057 (4)	0.0766 (19)	0.030*	0.5
H2B	0.532 (4)	0.134 (3)	0.052 (2)	0.030*	0.5
O3W	0.9929 (3)	0.0517 (3)	0.04517 (16)	0.0153 (7)	0.360 (4)
H3A	0.924 (4)	0.041 (6)	0.088 (2)	0.023*	0.360 (4)
H3B	1.020 (6)	0.136 (2)	0.052 (3)	0.023*	0.360 (4)
O4W	0.46051 (12)	0.06246 (10)	0.34209 (6)	0.0185 (2)	
H4A	0.489 (2)	−0.0103 (14)	0.3143 (11)	0.028*	
H4B	0.415 (2)	0.037 (2)	0.3893 (7)	0.028*	
O5W	0.0297 (3)	0.5553 (3)	0.50806 (17)	0.0403 (7)	0.5

### Tripotassium orthovanadate 0.56-hydrate (K3VO4_0.56H2O) Atomic displacement parameters (Å^2^)

**Table:**

	*U*^11^	*U*^22^	*U*^33^	*U*^12^	*U*^13^	*U*^23^
K1	0.00710 (9)	0.00880 (10)	0.01241 (10)	−0.00068 (7)	−0.00272 (8)	0.00052 (8)
K2	0.00841 (10)	0.00872 (10)	0.01068 (10)	−0.00143 (8)	−0.00065 (8)	−0.00214 (8)
K3	0.00752 (9)	0.01010 (10)	0.00941 (10)	−0.00116 (8)	−0.00196 (7)	−0.00004 (8)
K4	0.00733 (9)	0.00974 (10)	0.01259 (10)	−0.00094 (8)	−0.00096 (8)	−0.00327 (8)
K5	0.00783 (10)	0.01439 (11)	0.00890 (10)	0.00208 (8)	−0.00278 (8)	−0.00064 (8)
K6	0.00969 (10)	0.01077 (10)	0.00846 (9)	−0.00096 (8)	−0.00094 (8)	0.00105 (8)
K7A	0.01022 (18)	0.0144 (4)	0.0082 (4)	0.0012 (3)	−0.0001 (2)	−0.0007 (2)
K7B	0.01022 (18)	0.0144 (4)	0.0082 (4)	0.0012 (3)	−0.0001 (2)	−0.0007 (2)
K8A	0.0141 (4)	0.01030 (11)	0.00880 (11)	0.00119 (14)	−0.00052 (16)	0.00061 (9)
K8B	0.0141 (4)	0.01030 (11)	0.00880 (11)	0.00119 (14)	−0.00052 (16)	0.00061 (9)
K9	0.00786 (10)	0.01038 (10)	0.01675 (11)	0.00051 (8)	−0.00381 (8)	−0.00255 (9)
K10	0.00761 (9)	0.00866 (10)	0.01207 (10)	−0.00121 (8)	−0.00003 (8)	0.00013 (8)
K11	0.00809 (9)	0.00880 (10)	0.01051 (10)	0.00021 (7)	−0.00311 (8)	−0.00171 (8)
K12	0.00688 (9)	0.00944 (10)	0.00844 (9)	0.00004 (7)	−0.00205 (7)	−0.00036 (8)
K13	0.00863 (10)	0.00860 (10)	0.01484 (11)	−0.00033 (8)	0.00105 (8)	0.00124 (8)
K14	0.00704 (9)	0.00937 (10)	0.01003 (10)	−0.00048 (7)	−0.00028 (7)	0.00089 (8)
K15	0.01482 (11)	0.01375 (11)	0.00844 (10)	0.00432 (9)	−0.00089 (8)	−0.00062 (8)
K16	0.01581 (12)	0.01274 (11)	0.01095 (10)	−0.00134 (9)	−0.00362 (9)	−0.00312 (9)
K17	0.01672 (12)	0.01077 (11)	0.01148 (10)	−0.00246 (9)	−0.00455 (9)	−0.00109 (8)
K18	0.00799 (10)	0.02549 (14)	0.00998 (10)	−0.00288 (9)	−0.00207 (8)	0.00314 (10)
V1	0.00538 (7)	0.00640 (7)	0.00680 (7)	−0.00039 (6)	−0.00147 (6)	0.00070 (6)
V2	0.00643 (7)	0.00583 (7)	0.00596 (7)	−0.00062 (6)	−0.00201 (6)	0.00011 (6)
V3	0.00621 (7)	0.00603 (7)	0.00906 (8)	0.00048 (6)	−0.00314 (6)	−0.00152 (6)
V4	0.00577 (7)	0.00642 (8)	0.00685 (7)	−0.00039 (6)	−0.00172 (6)	−0.00009 (6)
V5	0.00534 (7)	0.00629 (7)	0.00512 (7)	−0.00094 (6)	−0.00084 (6)	−0.00036 (6)
V6	0.00537 (7)	0.00565 (7)	0.00559 (7)	0.00000 (6)	−0.00048 (6)	0.00004 (6)
O1	0.0090 (4)	0.0111 (4)	0.0081 (3)	−0.0009 (3)	0.0004 (3)	0.0003 (3)
O2	0.0082 (3)	0.0095 (4)	0.0089 (3)	−0.0014 (3)	−0.0011 (3)	0.0006 (3)
O3	0.0085 (4)	0.0283 (5)	0.0109 (4)	−0.0010 (4)	−0.0022 (3)	0.0089 (4)
O4	0.0156 (4)	0.0224 (5)	0.0143 (4)	−0.0074 (4)	−0.0087 (3)	0.0096 (4)
O5	0.0117 (4)	0.0085 (4)	0.0391 (6)	−0.0026 (3)	−0.0087 (4)	0.0025 (4)
O6	0.0103 (4)	0.0131 (4)	0.0195 (4)	−0.0015 (3)	−0.0067 (3)	0.0063 (3)
O7	0.0091 (4)	0.0107 (4)	0.0120 (4)	0.0021 (3)	−0.0032 (3)	0.0002 (3)
O8	0.0111 (4)	0.0138 (4)	0.0147 (4)	0.0011 (3)	−0.0004 (3)	−0.0070 (3)
O9	0.0093 (4)	0.0079 (4)	0.0140 (4)	−0.0020 (3)	−0.0013 (3)	−0.0002 (3)
O10	0.0135 (4)	0.0093 (4)	0.0245 (5)	−0.0013 (3)	−0.0082 (4)	−0.0032 (3)
O11	0.0085 (4)	0.0088 (4)	0.0159 (4)	0.0008 (3)	−0.0017 (3)	−0.0024 (3)
O12	0.0078 (4)	0.0112 (4)	0.0140 (4)	−0.0015 (3)	−0.0023 (3)	−0.0043 (3)
O13	0.0144 (4)	0.0089 (4)	0.0133 (4)	0.0014 (3)	0.0038 (3)	−0.0014 (3)
O14	0.0120 (4)	0.0139 (4)	0.0102 (4)	−0.0016 (3)	−0.0012 (3)	−0.0047 (3)
O15	0.0090 (4)	0.0141 (4)	0.0083 (3)	−0.0004 (3)	−0.0015 (3)	−0.0033 (3)
O16	0.0115 (4)	0.0138 (4)	0.0108 (4)	0.0050 (3)	−0.0042 (3)	−0.0055 (3)
O17	0.0086 (4)	0.0120 (4)	0.0079 (3)	−0.0006 (3)	−0.0026 (3)	0.0014 (3)
O18	0.0096 (4)	0.0122 (4)	0.0094 (4)	0.0008 (3)	0.0000 (3)	−0.0018 (3)
O19	0.0079 (3)	0.0117 (4)	0.0089 (3)	0.0015 (3)	0.0003 (3)	−0.0018 (3)
O20	0.0101 (4)	0.0165 (4)	0.0123 (4)	−0.0030 (3)	−0.0017 (3)	0.0072 (3)
O21	0.0100 (4)	0.0129 (4)	0.0076 (3)	−0.0016 (3)	−0.0019 (3)	−0.0013 (3)
O22	0.0090 (4)	0.0089 (4)	0.0100 (4)	0.0008 (3)	−0.0018 (3)	0.0019 (3)
O23	0.0131 (4)	0.0097 (4)	0.0131 (4)	−0.0041 (3)	−0.0044 (3)	0.0018 (3)
O24	0.0100 (4)	0.0103 (4)	0.0145 (4)	−0.0022 (3)	−0.0042 (3)	−0.0026 (3)
O1W	0.0166 (4)	0.0187 (5)	0.0121 (4)	0.0026 (4)	0.0022 (3)	−0.0008 (4)
O2W	0.0156 (9)	0.0235 (11)	0.0206 (10)	−0.0013 (8)	−0.0029 (8)	−0.0062 (8)
O3W	0.0108 (12)	0.0194 (14)	0.0160 (13)	0.0007 (9)	−0.0024 (9)	−0.0057 (10)
O4W	0.0264 (5)	0.0132 (4)	0.0130 (4)	−0.0034 (4)	0.0062 (4)	−0.0022 (3)
O5W	0.0526 (19)	0.0262 (13)	0.0339 (14)	0.0075 (13)	0.0137 (13)	−0.0006 (11)

### Tripotassium orthovanadate 0.56-hydrate (K3VO4_0.56H2O) Geometric parameters (Å, º)

**Table:**

K1—O4	2.6147 (10)	K10—O20	2.7890 (10)
K1—O5	2.7147 (10)	K10—O14^xi^	2.7998 (9)
K1—O1	2.7347 (9)	K10—O3	2.8029 (10)
K1—O9^i^	2.7420 (10)	K10—O24^xiii^	2.8658 (10)
K1—O22^ii^	2.7686 (9)	K10—O2W	3.087 (2)
K1—O5W^i^	3.050 (3)	K10—O10	3.2499 (11)
K1—O10	3.1147 (11)	K10—V5^xiii^	3.3732 (3)
K1—V6^ii^	3.3523 (3)	K10—V5	3.4193 (3)
K1—K9	3.3993 (4)	K10—V1	3.4379 (3)
K1—K5^i^	3.5058 (4)	K10—H2B	2.73 (4)
K1—V1	3.5170 (4)	K11—O18^v^	2.6906 (10)
K1—K6	3.6584 (4)	K11—O11^iii^	2.7388 (10)
K2—O8	2.5976 (10)	K11—O7	2.8167 (10)
K2—O6^i^	2.6489 (10)	K11—O17	2.8325 (9)
K2—O7	2.7329 (9)	K11—O16	2.8560 (10)
K2—O11^iii^	2.7893 (10)	K11—O14^v^	2.8956 (10)
K2—O1^iii^	2.8007 (10)	K11—O8	2.9803 (10)
K2—O3W^iv^	2.937 (3)	K11—V4^v^	3.3830 (3)
K2—O23^iv^	2.9519 (10)	K11—V3	3.4180 (4)
K2—V1^iii^	3.2645 (3)	K11—V5	3.4215 (3)
K2—V4^i^	3.4006 (3)	K12—O12	2.7139 (9)
K2—K11	3.5165 (4)	K12—O16^v^	2.7195 (10)
K2—K16^iv^	3.5890 (4)	K12—O15^v^	2.7649 (9)
K2—K17^v^	3.5892 (4)	K12—O18	2.7747 (10)
K3—O24^v^	2.6578 (9)	K12—O9	2.7765 (10)
K3—O3^vi^	2.7388 (10)	K12—O6	2.9877 (10)
K3—O8^i^	2.8086 (10)	K12—O19^v^	3.0687 (10)
K3—O1W	2.8533 (11)	K12—V3^v^	3.3233 (3)
K3—O14	2.8963 (10)	K12—V6^v^	3.4143 (3)
K3—O24^vii^	2.8978 (10)	K12—V4	3.4344 (4)
K3—V5^i^	3.4803 (3)	K12—K14	3.5294 (4)
K3—K10^vi^	3.5075 (4)	K13—O20	2.6615 (9)
K3—V4	3.7117 (4)	K13—O21	2.7231 (9)
K3—K3^viii^	3.7292 (5)	K13—O4W	2.7912 (11)
K3—K18^vii^	3.7529 (4)	K13—O18^xiv^	2.8199 (10)
K3—H1A	2.72 (2)	K13—O11	2.8326 (10)
K4—O9	2.6509 (9)	K13—O10	2.9148 (10)
K4—O6	2.6908 (10)	K13—O2W	2.942 (2)
K4—O7^i^	2.7028 (10)	K13—O23	2.9768 (10)
K4—O12	2.7263 (10)	K13—V4^xiv^	3.4711 (4)
K4—O5^i^	2.7796 (11)	K13—V1	3.5064 (4)
K4—O2	2.8147 (9)	K13—K18	3.6122 (4)
K4—V2	3.2901 (3)	K13—H2A	2.69 (4)
K4—V3^i^	3.3150 (3)	K13—H2B	2.47 (4)
K4—O1W	3.3424 (11)	K13—H4A	2.67 (2)
K4—K12	3.4572 (4)	K14—O13^xiv^	2.5959 (10)
K4—H1A	2.97 (2)	K14—O12	2.6799 (9)
K4—H1B	2.94 (2)	K14—O22	2.7416 (9)
K5—O22^v^	2.5843 (9)	K14—O15^v^	2.8066 (9)
K5—O2^i^	2.7106 (9)	K14—O19	2.8195 (10)
K5—O4^i^	2.7465 (11)	K14—O13	2.9085 (10)
K5—O15	2.7626 (9)	K14—O4W	3.2304 (11)
K5—O13^iii^	2.7961 (10)	K14—V6	3.3553 (3)
K5—O2^iii^	2.9417 (9)	K14—V2	3.3748 (3)
K5—O5W	3.159 (3)	K14—H4B	2.90 (2)
K5—V2^i^	3.3285 (3)	K15—O16	2.7112 (10)
K5—K17^v^	3.3314 (4)	K15—O21	2.8031 (10)
K5—V2^iii^	3.3741 (4)	K15—O17	2.8241 (9)
K5—K14^v^	3.3974 (4)	K15—O24	2.9169 (10)
K6—O10	2.6495 (10)	K15—O14^v^	3.0747 (10)
K6—O17	2.7353 (9)	K15—O20	3.0799 (11)
K6—O1W^i^	2.7955 (11)	K15—O1W^v^	3.3022 (11)
K6—O16	2.9221 (10)	K15—V5^xiii^	3.5038 (4)
K6—O7	3.0708 (10)	K15—K18	3.5437 (4)
K6—O8	3.0826 (11)	K16—O22	2.5582 (10)
K6—V3	3.2430 (3)	K16—O2^xiv^	2.8481 (9)
K6—V5	3.2670 (3)	K16—O1^xv^	2.8961 (9)
K6—O5	3.2746 (13)	K16—O23	2.9735 (10)
K6—K11	3.5686 (4)	K16—O6^xiv^	3.1851 (11)
K6—K10	3.6592 (4)	K16—O18^xiv^	3.1992 (10)
K7A—K7B^ix^	0.3646 (12)	K16—O4W	3.2150 (12)
K7A—O3W^ii^	2.569 (3)	K16—V4^xiv^	3.2872 (3)
K7A—O2W	2.643 (3)	K16—O21	3.4079 (10)
K7A—O2W^ix^	2.749 (3)	K16—V6	3.5826 (4)
K7A—O11	2.8028 (16)	K16—V2^xiv^	3.6715 (4)
K7A—O3W^ix^	2.824 (3)	K16—H4A	3.03 (2)
K7A—O23^ix^	2.8455 (16)	K17—O7^v^	2.6822 (10)
K7A—O8^x^	2.9053 (17)	K17—O4^xiv^	2.8205 (12)
K7A—O3	2.9300 (18)	K17—O1^xiv^	2.8729 (10)
K7A—O14^xi^	2.9454 (16)	K17—O2^xv^	2.9425 (9)
K7A—O20^ix^	3.2662 (17)	K17—O4W^xiv^	2.9598 (12)
K7A—V4^xi^	3.4847 (13)	K17—O13^xiv^	3.1162 (11)
K7A—H2A	2.28 (4)	K17—O11^xiv^	3.3020 (10)
K7B—O2W	2.605 (3)	K17—O15^v^	3.3897 (10)
K7B—O23	2.6286 (19)	K17—V1^xiv^	3.5014 (4)
K7B—O3W^xii^	2.728 (3)	K17—V2^xiv^	3.5718 (3)
K7B—O3W	2.731 (3)	K18—O21	2.7547 (9)
K7B—O2W^ix^	2.759 (3)	K18—O3^xv^	2.7660 (10)
K7B—O8^xiii^	2.765 (2)	K18—O23	2.8203 (10)
K7B—O20	2.9298 (18)	K18—O1^xv^	2.8827 (10)
K7B—O14^xiv^	3.026 (2)	K18—O20	2.9690 (10)
K7B—O11^ix^	3.0665 (18)	K18—O3W	3.030 (3)
K7B—O3^ix^	3.2834 (18)	K18—O24	3.0914 (10)
K7B—V4^xiv^	3.3966 (18)	K18—O1W^v^	3.1367 (12)
K7B—H2B	2.43 (5)	K18—V1^xv^	3.4229 (4)
K7B—H3A	2.51 (6)	K18—V5^xiii^	3.4545 (4)
K7B—H2A^ix^	2.48 (4)	K18—H3A	2.58 (6)
K8A—O12	2.6766 (11)	K18—H3B	2.52 (5)
K8A—O19	2.7411 (13)	V1—O10	1.7109 (10)
K8A—O19^v^	2.7674 (16)	V1—O11	1.7212 (9)
K8A—O5W	2.804 (4)	V1—O3	1.7216 (10)
K8A—O15	2.8682 (11)	V1—O1	1.7227 (9)
K8A—O5	3.0080 (17)	V2—O12	1.7116 (9)
K8A—O9	3.2290 (10)	V2—O4	1.7139 (10)
K8A—V6^v^	3.3406 (8)	V2—O13	1.7214 (9)
K8A—K9	3.4411 (6)	V2—O2	1.7220 (9)
K8A—V3	3.4677 (8)	V3—O16	1.7062 (9)
K8A—K12	3.7811 (9)	V3—O15	1.7118 (9)
K8A—K14^v^	3.7982 (9)	V3—O7	1.7258 (9)
K8B—O5W	2.463 (8)	V3—O5	1.7379 (10)
K8B—O12	2.696 (4)	V4—O14	1.7094 (9)
K8B—O5	2.729 (6)	V4—O18	1.7139 (9)
K8B—O15	2.855 (4)	V4—O23^xiv^	1.7169 (9)
K8B—O19	2.976 (6)	V4—O6	1.7299 (9)
K8B—O19^v^	3.080 (7)	V5—O24^xiii^	1.7112 (9)
K8B—O5W^i^	3.110 (9)	V5—O8	1.7142 (9)
K8B—O9	3.282 (4)	V5—O17	1.7180 (9)
K8B—V3	3.332 (4)	V5—O20^xiii^	1.7231 (9)
K8B—K9	3.374 (4)	V6—O22	1.7096 (9)
K8B—V6^v^	3.508 (5)	V6—O19	1.7141 (9)
K8B—V2	3.789 (4)	V6—O21	1.7151 (9)
K9—O10	2.6385 (11)	V6—O9^v^	1.7282 (9)
K9—O19	2.6519 (10)	O1W—H1A	0.944 (9)
K9—O5	2.8308 (11)	O1W—H1B	0.939 (9)
K9—O21	2.8855 (10)	O2W—H2A	0.966 (10)
K9—O16	2.9345 (10)	O2W—H2B	0.964 (10)
K9—O4	2.9666 (10)	O3W—H3A	0.958 (10)
K9—O4W	3.0139 (11)	O3W—H3B	0.957 (10)
K9—V6	3.3361 (3)	O4W—H4A	0.937 (9)
K9—K13	3.4053 (4)	O4W—H4B	0.931 (9)
K10—O17^xiii^	2.7573 (9)	O5W—O5W^i^	1.393 (6)
K10—O17	2.7873 (10)		
			
O4—K1—O5	86.09 (3)	V6^v^—K12—K14	116.514 (9)
O4—K1—O1	95.53 (3)	V4—K12—K14	121.424 (10)
O5—K1—O1	140.00 (3)	K4—K12—K14	98.891 (9)
O4—K1—O9^i^	141.56 (3)	O20—K13—O21	85.44 (3)
O5—K1—O9^i^	92.25 (3)	O20—K13—O4W	161.70 (3)
O1—K1—O9^i^	109.49 (3)	O21—K13—O4W	76.51 (3)
O4—K1—O22^ii^	96.40 (3)	O20—K13—O18^xiv^	130.13 (3)
O5—K1—O22^ii^	137.00 (3)	O21—K13—O18^xiv^	112.05 (3)
O1—K1—O22^ii^	82.67 (3)	O4W—K13—O18^xiv^	56.85 (3)
O9^i^—K1—O22^ii^	60.05 (3)	O20—K13—O11	102.33 (3)
O4—K1—O5W^i^	65.56 (7)	O21—K13—O11	150.35 (3)
O5—K1—O5W^i^	60.07 (7)	O4W—K13—O11	94.95 (3)
O1—K1—O5W^i^	153.94 (6)	O18^xiv^—K13—O11	84.94 (3)
O9^i^—K1—O5W^i^	80.42 (7)	O20—K13—O10	92.49 (3)
O22^ii^—K1—O5W^i^	81.94 (7)	O21—K13—O10	93.31 (3)
O4—K1—O10	92.49 (3)	O4W—K13—O10	91.54 (3)
O5—K1—O10	82.75 (3)	O18^xiv^—K13—O10	130.14 (3)
O1—K1—O10	57.26 (3)	O11—K13—O10	58.15 (3)
O9^i^—K1—O10	125.44 (3)	O20—K13—O2W	55.71 (5)
O22^ii^—K1—O10	139.63 (3)	O21—K13—O2W	141.10 (5)
O5W^i^—K1—O10	136.73 (7)	O4W—K13—O2W	142.09 (5)
O4—K1—V6^ii^	125.14 (2)	O18^xiv^—K13—O2W	94.22 (5)
O5—K1—V6^ii^	120.88 (2)	O11—K13—O2W	55.18 (5)
O1—K1—V6^ii^	90.55 (2)	O10—K13—O2W	90.57 (5)
O9^i^—K1—V6^ii^	30.928 (19)	O20—K13—O23	78.35 (3)
O22^ii^—K1—V6^ii^	30.583 (19)	O21—K13—O23	88.14 (3)
O5W^i^—K1—V6^ii^	86.81 (7)	O4W—K13—O23	97.83 (3)
O10—K1—V6^ii^	134.09 (2)	O18^xiv^—K13—O23	57.17 (3)
O4—K1—K9	57.35 (2)	O11—K13—O23	121.36 (3)
O5—K1—K9	53.76 (2)	O10—K13—O23	170.59 (3)
O1—K1—K9	93.99 (2)	O2W—K13—O23	82.53 (5)
O9^i^—K1—K9	143.89 (2)	O20—K13—K9	114.11 (2)
O22^ii^—K1—K9	153.24 (2)	O21—K13—K9	54.82 (2)
O5W^i^—K1—K9	90.39 (7)	O4W—K13—K9	57.18 (2)
O10—K1—K9	47.540 (19)	O18^xiv^—K13—K9	113.88 (2)
V6^ii^—K1—K9	174.596 (10)	O11—K13—K9	96.53 (2)
O4—K1—K5^i^	50.81 (2)	O10—K13—K9	48.59 (2)
O5—K1—K5^i^	113.78 (3)	O2W—K13—K9	139.02 (5)
O1—K1—K5^i^	97.25 (2)	O23—K13—K9	137.47 (2)
O9^i^—K1—K5^i^	96.19 (2)	O20—K13—V4^xiv^	101.55 (2)
O22^ii^—K1—K5^i^	46.848 (19)	O21—K13—V4^xiv^	108.13 (2)
O5W^i^—K1—K5^i^	57.10 (6)	O4W—K13—V4^xiv^	81.58 (2)
O10—K1—K5^i^	135.43 (2)	O18^xiv^—K13—V4^xiv^	29.355 (19)
V6^ii^—K1—K5^i^	74.329 (8)	O11—K13—V4^xiv^	98.38 (2)
K9—K1—K5^i^	107.939 (10)	O10—K13—V4^xiv^	155.07 (2)
O4—K1—V1	98.50 (3)	O2W—K13—V4^xiv^	80.77 (5)
O5—K1—V1	111.49 (3)	O23—K13—V4^xiv^	29.636 (18)
O1—K1—V1	28.653 (19)	K9—K13—V4^xiv^	137.122 (11)
O9^i^—K1—V1	117.54 (2)	O20—K13—V1	97.41 (2)
O22^ii^—K1—V1	110.53 (2)	O21—K13—V1	122.19 (2)
O5W^i^—K1—V1	161.39 (7)	O4W—K13—V1	94.77 (3)
O10—K1—V1	29.098 (18)	O18^xiv^—K13—V1	109.21 (2)
V6^ii^—K1—V1	111.038 (9)	O11—K13—V1	29.107 (19)
K9—K1—V1	72.240 (8)	O10—K13—V1	29.082 (19)
K5^i^—K1—V1	121.160 (10)	O2W—K13—V1	70.47 (4)
O4—K1—K6	124.71 (2)	O23—K13—V1	149.22 (2)
O5—K1—K6	59.67 (3)	K9—K13—V1	72.302 (8)
O1—K1—K6	88.02 (2)	V4^xiv^—K13—V1	127.235 (9)
O9^i^—K1—K6	85.92 (2)	O20—K13—K18	53.95 (2)
O22^ii^—K1—K6	138.57 (2)	O21—K13—K18	49.12 (2)
O5W^i^—K1—K6	117.23 (6)	O4W—K13—K18	110.04 (3)
O10—K1—K6	45.175 (19)	O18^xiv^—K13—K18	102.13 (2)
V6^ii^—K1—K6	109.922 (9)	O11—K13—K18	153.89 (2)
K9—K1—K6	67.365 (9)	O10—K13—K18	126.05 (2)
K5^i^—K1—K6	173.278 (10)	O2W—K13—K18	98.95 (4)
V1—K1—K6	62.802 (8)	O23—K13—K18	49.540 (19)
O8—K2—O6^i^	105.02 (3)	K9—K13—K18	103.212 (10)
O8—K2—O7	90.95 (3)	V4^xiv^—K13—K18	78.613 (9)
O6^i^—K2—O7	89.84 (3)	V1—K13—K18	147.397 (10)
O8—K2—O11^iii^	85.45 (3)	O20—K13—H2A	70.8 (6)
O6^i^—K2—O11^iii^	168.05 (3)	O21—K13—H2A	153.9 (9)
O7—K2—O11^iii^	84.14 (3)	O4W—K13—H2A	127.5 (6)
O8—K2—O1^iii^	142.26 (3)	O18^xiv^—K13—H2A	92.1 (10)
O6^i^—K2—O1^iii^	111.14 (3)	O11—K13—H2A	36.1 (2)
O7—K2—O1^iii^	99.47 (3)	O10—K13—H2A	77.5 (7)
O11^iii^—K2—O1^iii^	60.03 (3)	O2W—K13—H2A	19.1 (2)
O8—K2—O3W^iv^	80.21 (6)	O23—K13—H2A	97.2 (7)
O6^i^—K2—O3W^iv^	106.09 (6)	K9—K13—H2A	125.3 (6)
O7—K2—O3W^iv^	163.29 (6)	V4^xiv^—K13—H2A	87.7 (9)
O11^iii^—K2—O3W^iv^	81.05 (6)	V1—K13—H2A	53.4 (5)
O1^iii^—K2—O3W^iv^	79.77 (6)	K18—K13—H2A	117.9 (3)
O8—K2—O23^iv^	115.01 (3)	O20—K13—H2B	37.8 (4)
O6^i^—K2—O23^iv^	59.33 (3)	O21—K13—H2B	123.2 (4)
O7—K2—O23^iv^	143.06 (3)	O4W—K13—H2B	159.8 (5)
O11^iii^—K2—O23^iv^	121.79 (3)	O18^xiv^—K13—H2B	107.0 (8)
O1^iii^—K2—O23^iv^	76.39 (3)	O11—K13—H2B	70.0 (7)
O3W^iv^—K2—O23^iv^	53.28 (6)	O10—K13—H2B	91.5 (11)
O8—K2—V1^iii^	110.49 (2)	O2W—K13—H2B	18.0 (4)
O6^i^—K2—V1^iii^	141.88 (2)	O23—K13—H2B	80.0 (11)
O7—K2—V1^iii^	102.79 (2)	K9—K13—H2B	135.5 (10)
O11^iii^—K2—V1^iii^	31.819 (19)	V4^xiv^—K13—H2B	87.3 (10)
O1^iii^—K2—V1^iii^	31.846 (18)	V1—K13—H2B	78.6 (10)
O3W^iv^—K2—V1^iii^	67.77 (6)	K18—K13—H2B	83.9 (7)
O23^iv^—K2—V1^iii^	92.55 (2)	H2A—K13—H2B	34.7 (7)
O8—K2—V4^i^	107.22 (2)	O20—K13—H4A	162.3 (4)
O6^i^—K2—V4^i^	30.09 (2)	O21—K13—H4A	90.2 (4)
O7—K2—V4^i^	119.47 (2)	O4W—K13—H4A	19.6 (2)
O11^iii^—K2—V4^i^	152.08 (2)	O18^xiv^—K13—H4A	37.4 (2)
O1^iii^—K2—V4^i^	98.94 (2)	O11—K13—H4A	89.9 (5)
O3W^iv^—K2—V4^i^	76.92 (6)	O10—K13—H4A	104.9 (4)
O23^iv^—K2—V4^i^	30.323 (18)	O2W—K13—H4A	126.0 (3)
V1^iii^—K2—V4^i^	121.650 (10)	O23—K13—H4A	84.4 (4)
O8—K2—K11	55.94 (2)	K9—K13—H4A	76.5 (2)
O6^i^—K2—K11	131.86 (2)	V4^xiv^—K13—H4A	63.6 (3)
O7—K2—K11	51.74 (2)	V1—K13—H4A	99.4 (5)
O11^iii^—K2—K11	49.87 (2)	K18—K13—H4A	111.1 (5)
O1^iii^—K2—K11	103.79 (2)	H2A—K13—H4A	115.7 (9)
O3W^iv^—K2—K11	111.96 (5)	H2B—K13—H4A	142.2 (7)
O23^iv^—K2—K11	165.19 (2)	O13^xiv^—K14—O12	129.79 (3)
V1^iii^—K2—K11	81.278 (9)	O13^xiv^—K14—O22	86.90 (3)
V4^i^—K2—K11	156.696 (10)	O12—K14—O22	143.29 (3)
O8—K2—K16^iv^	162.92 (2)	O13^xiv^—K14—O15^v^	79.65 (3)
O6^i^—K2—K16^iv^	59.14 (2)	O12—K14—O15^v^	99.24 (3)
O7—K2—K16^iv^	95.09 (2)	O22—K14—O15^v^	87.37 (3)
O11^iii^—K2—K16^iv^	111.03 (2)	O13^xiv^—K14—O19	146.55 (3)
O1^iii^—K2—K16^iv^	52.142 (19)	O12—K14—O19	83.62 (3)
O3W^iv^—K2—K16^iv^	97.43 (5)	O22—K14—O19	59.76 (3)
O23^iv^—K2—K16^iv^	53.00 (2)	O15^v^—K14—O19	95.12 (3)
V1^iii^—K2—K16^iv^	83.747 (9)	O13^xiv^—K14—O13	89.27 (3)
V4^i^—K2—K16^iv^	56.030 (7)	O12—K14—O13	60.06 (3)
K11—K2—K16^iv^	138.516 (10)	O22—K14—O13	129.86 (3)
O8—K2—K17^v^	125.82 (2)	O15^v^—K14—O13	140.73 (3)
O6^i^—K2—K17^v^	107.63 (2)	O19—K14—O13	113.31 (3)
O7—K2—K17^v^	47.88 (2)	O13^xiv^—K14—O4W	94.21 (3)
O11^iii^—K2—K17^v^	60.83 (2)	O12—K14—O4W	94.09 (3)
O1^iii^—K2—K17^v^	51.65 (2)	O22—K14—O4W	80.05 (3)
O3W^iv^—K2—K17^v^	128.08 (6)	O15^v^—K14—O4W	166.32 (3)
O23^iv^—K2—K17^v^	118.67 (2)	O19—K14—O4W	83.24 (3)
V1^iii^—K2—K17^v^	61.228 (8)	O13—K14—O4W	50.41 (3)
V4^i^—K2—K17^v^	122.571 (9)	O13^xiv^—K14—V6	116.80 (2)
K11—K2—K17^v^	70.051 (9)	O12—K14—V6	113.03 (2)
K16^iv^—K2—K17^v^	68.808 (8)	O22—K14—V6	30.502 (19)
O24^v^—K3—O3^vi^	90.11 (3)	O15^v^—K14—V6	98.27 (2)
O24^v^—K3—O8^i^	150.03 (3)	O19—K14—V6	30.695 (18)
O3^vi^—K3—O8^i^	107.52 (3)	O13—K14—V6	120.13 (2)
O24^v^—K3—O1W	78.91 (3)	O4W—K14—V6	73.45 (2)
O3^vi^—K3—O1W	160.33 (3)	O13^xiv^—K14—V2	114.97 (2)
O8^i^—K3—O1W	89.54 (3)	O12—K14—V2	30.15 (2)
O24^v^—K3—O14	86.05 (3)	O22—K14—V2	141.65 (2)
O3^vi^—K3—O14	81.29 (3)	O15^v^—K14—V2	125.83 (2)
O8^i^—K3—O14	119.83 (3)	O19—K14—V2	95.00 (2)
O1W—K3—O14	81.71 (3)	O13—K14—V2	30.668 (19)
O24^v^—K3—O24^vii^	95.79 (3)	O4W—K14—V2	67.84 (2)
O3^vi^—K3—O24^vii^	98.40 (3)	V6—K14—V2	116.037 (9)
O8^i^—K3—O24^vii^	58.48 (3)	O13^xiv^—K14—K5^v^	53.62 (2)
O1W—K3—O24^vii^	98.89 (3)	O12—K14—K5^v^	151.05 (2)
O14—K3—O24^vii^	178.14 (3)	O22—K14—K5^v^	48.352 (19)
O24^v^—K3—V5^i^	124.28 (2)	O15^v^—K14—K5^v^	51.82 (2)
O3^vi^—K3—V5^i^	103.17 (2)	O19—K14—K5^v^	97.25 (2)
O8^i^—K3—V5^i^	29.219 (19)	O13—K14—K5^v^	141.27 (2)
O1W—K3—V5^i^	96.49 (2)	O4W—K14—K5^v^	114.79 (2)
O14—K3—V5^i^	148.91 (2)	V6—K14—K5^v^	75.726 (8)
O24^vii^—K3—V5^i^	29.350 (18)	V2—K14—K5^v^	167.691 (10)
O24^v^—K3—K10^vi^	53.26 (2)	O13^xiv^—K14—K12	106.27 (2)
O3^vi^—K3—K10^vi^	51.55 (2)	O12—K14—K12	49.55 (2)
O8^i^—K3—K10^vi^	155.11 (2)	O22—K14—K12	129.97 (2)
O1W—K3—K10^vi^	109.36 (2)	O15^v^—K14—K12	50.172 (19)
O14—K3—K10^vi^	50.759 (19)	O19—K14—K12	94.66 (2)
O24^vii^—K3—K10^vi^	130.35 (2)	O13—K14—K12	98.98 (2)
V5^i^—K3—K10^vi^	151.605 (10)	O4W—K14—K12	143.42 (2)
O24^v^—K3—V4	106.42 (2)	V6—K14—K12	120.070 (9)
O3^vi^—K3—V4	98.02 (2)	V2—K14—K12	76.029 (8)
O8^i^—K3—V4	95.16 (2)	K5^v^—K14—K12	101.729 (9)
O1W—K3—V4	70.08 (2)	O13^xiv^—K14—K8A^v^	117.85 (3)
O14—K3—V4	26.490 (18)	O12—K14—K8A^v^	94.99 (3)
O24^vii^—K3—V4	152.23 (2)	O22—K14—K8A^v^	62.66 (2)
V5^i^—K3—V4	124.207 (9)	O15^v^—K14—K8A^v^	48.68 (3)
K10^vi^—K3—V4	77.178 (8)	O19—K14—K8A^v^	46.59 (2)
O24^v^—K3—K3^viii^	50.63 (2)	O13—K14—K8A^v^	152.14 (3)
O3^vi^—K3—K3^viii^	96.60 (3)	O4W—K14—K8A^v^	127.25 (2)
O8^i^—K3—K3^viii^	102.19 (2)	V6—K14—K8A^v^	55.259 (9)
O1W—K3—K3^viii^	89.03 (2)	V2—K14—K8A^v^	122.36 (2)
O14—K3—K3^viii^	136.68 (2)	K5^v^—K14—K8A^v^	66.57 (2)
O24^vii^—K3—K3^viii^	45.158 (19)	K12—K14—K8A^v^	68.737 (10)
V5^i^—K3—K3^viii^	73.984 (9)	O13^xiv^—K14—H4B	90.6 (4)
K10^vi^—K3—K3^viii^	94.404 (10)	O12—K14—H4B	84.2 (4)
V4—K3—K3^viii^	152.746 (12)	O22—K14—H4B	95.8 (2)
O24^v^—K3—K18^vii^	107.10 (2)	O15^v^—K14—H4B	169.6 (4)
O3^vi^—K3—K18^vii^	47.32 (2)	O19—K14—H4B	95.0 (3)
O8^i^—K3—K18^vii^	71.47 (2)	O13—K14—H4B	34.3 (2)
O1W—K3—K18^vii^	151.78 (2)	O4W—K14—H4B	16.4 (2)
O14—K3—K18^vii^	125.64 (2)	V6—K14—H4B	89.3 (2)
O24^vii^—K3—K18^vii^	53.54 (2)	V2—K14—H4B	55.4 (3)
V5^i^—K3—K18^vii^	56.906 (7)	K5^v^—K14—H4B	124.3 (4)
K10^vi^—K3—K18^vii^	95.384 (9)	K12—K14—H4B	131.0 (3)
V4—K3—K18^vii^	130.675 (10)	K8A^v^—K14—H4B	141.2 (3)
K3^viii^—K3—K18^vii^	75.391 (10)	O16—K15—O21	84.87 (3)
O24^v^—K3—K2^i^	149.69 (2)	O16—K15—O17	79.69 (3)
O3^vi^—K3—K2^i^	113.10 (3)	O21—K15—O17	139.13 (3)
O8^i^—K3—K2^i^	42.12 (2)	O16—K15—O24	169.26 (3)
O1W—K3—K2^i^	73.01 (2)	O21—K15—O24	105.81 (3)
O14—K3—K2^i^	78.94 (2)	O17—K15—O24	92.52 (3)
O24^vii^—K3—K2^i^	99.536 (19)	O16—K15—O14^v^	92.50 (3)
V5^i^—K3—K2^i^	70.976 (7)	O21—K15—O14^v^	141.00 (3)
K10^vi^—K3—K2^i^	127.077 (9)	O17—K15—O14^v^	77.69 (3)
V4—K3—K2^i^	53.237 (7)	O24—K15—O14^v^	78.52 (3)
K3^viii^—K3—K2^i^	138.075 (12)	O16—K15—O20	130.20 (3)
K18^vii^—K3—K2^i^	103.045 (9)	O21—K15—O20	76.62 (3)
O24^v^—K3—H1A	91.5 (4)	O17—K15—O20	84.99 (3)
O3^vi^—K3—H1A	148.8 (3)	O24—K15—O20	55.49 (3)
O8^i^—K3—H1A	85.7 (5)	O14^v^—K15—O20	129.90 (3)
O1W—K3—H1A	19.3 (2)	O16—K15—O1W^v^	114.82 (3)
O14—K3—H1A	67.7 (4)	O21—K15—O1W^v^	73.77 (3)
O24^vii^—K3—H1A	112.4 (4)	O17—K15—O1W^v^	146.80 (3)
V5^i^—K3—H1A	101.6 (5)	O24—K15—O1W^v^	68.29 (3)
K10^vi^—K3—H1A	106.7 (5)	O14^v^—K15—O1W^v^	72.19 (3)
V4—K3—H1A	51.8 (3)	O20—K15—O1W^v^	103.72 (3)
K3^viii^—K3—H1A	108.3 (2)	O16—K15—V5^xiii^	151.84 (2)
K18^vii^—K3—H1A	157.0 (5)	O21—K15—V5^xiii^	99.62 (2)
K2^i^—K3—H1A	58.5 (4)	O17—K15—V5^xiii^	78.95 (2)
O9—K4—O6	81.02 (3)	O24—K15—V5^xiii^	29.122 (18)
O9—K4—O7^i^	132.70 (3)	O14^v^—K15—V5^xiii^	100.609 (19)
O6—K4—O7^i^	89.60 (3)	O20—K15—V5^xiii^	29.452 (17)
O9—K4—O12	83.20 (3)	O1W^v^—K15—V5^xiii^	92.992 (19)
O6—K4—O12	96.80 (3)	O16—K15—K18	134.38 (2)
O7^i^—K4—O12	144.09 (3)	O21—K15—K18	49.79 (2)
O9—K4—O5^i^	92.80 (3)	O17—K15—K18	136.12 (2)
O6—K4—O5^i^	133.88 (4)	O24—K15—K18	56.17 (2)
O7^i^—K4—O5^i^	61.62 (3)	O14^v^—K15—K18	118.38 (2)
O12—K4—O5^i^	128.02 (3)	O20—K15—K18	52.696 (19)
O9—K4—O2	143.86 (3)	O1W^v^—K15—K18	54.40 (2)
O6—K4—O2	99.10 (3)	V5^xiii^—K15—K18	58.700 (7)
O7^i^—K4—O2	83.31 (3)	O16—K15—K10^xiii^	118.01 (2)
O12—K4—O2	60.80 (3)	O21—K15—K10^xiii^	156.56 (2)
O5^i^—K4—O2	111.16 (3)	O17—K15—K10^xiii^	49.675 (19)
O9—K4—V2	113.67 (2)	O24—K15—K10^xiii^	51.48 (2)
O6—K4—V2	107.60 (2)	O14^v^—K15—K10^xiii^	49.361 (18)
O7^i^—K4—V2	113.37 (2)	O20—K15—K10^xiii^	83.918 (19)
O12—K4—V2	31.304 (19)	O1W^v^—K15—K10^xiii^	98.76 (2)
O5^i^—K4—V2	116.50 (3)	V5^xiii^—K15—K10^xiii^	57.987 (7)
O2—K4—V2	31.558 (18)	K18—K15—K10^xiii^	107.609 (10)
O9—K4—V3^i^	119.91 (2)	O16—K15—K11	50.97 (2)
O6—K4—V3^i^	117.70 (2)	O21—K15—K11	135.19 (2)
O7^i^—K4—V3^i^	31.272 (19)	O17—K15—K11	50.09 (2)
O12—K4—V3^i^	139.90 (2)	O24—K15—K11	118.30 (2)
O5^i^—K4—V3^i^	31.61 (2)	O14^v^—K15—K11	50.270 (19)
O2—K4—V3^i^	92.28 (2)	O20—K15—K11	135.08 (2)
V2—K4—V3^i^	112.797 (9)	O1W^v^—K15—K11	114.49 (2)
O9—K4—O1W	49.92 (3)	V5^xiii^—K15—K11	122.579 (10)
O6—K4—O1W	50.68 (3)	K18—K15—K11	168.214 (12)
O7^i^—K4—O1W	88.93 (3)	K10^xiii^—K15—K11	68.228 (8)
O12—K4—O1W	122.12 (3)	O16—K15—K12^v^	45.99 (2)
O5^i^—K4—O1W	90.95 (3)	O21—K15—K12^v^	75.16 (2)
O2—K4—O1W	148.96 (3)	O17—K15—K12^v^	116.49 (2)
V2—K4—O1W	150.28 (2)	O24—K15—K12^v^	134.85 (2)
V3^i^—K4—O1W	96.51 (2)	O14^v^—K15—K12^v^	75.298 (18)
O9—K4—K12	52.06 (2)	O20—K15—K12^v^	151.77 (2)
O6—K4—K12	56.51 (2)	O1W^v^—K15—K12^v^	68.970 (19)
O7^i^—K4—K12	145.95 (2)	V5^xiii^—K15—K12^v^	161.943 (10)
O12—K4—K12	50.38 (2)	K18—K15—K12^v^	107.258 (10)
O5^i^—K4—K12	144.06 (2)	K10^xiii^—K15—K12^v^	123.668 (10)
O2—K4—K12	97.76 (2)	K11—K15—K12^v^	68.614 (8)
V2—K4—K12	78.121 (8)	O22—K16—O2^xiv^	89.92 (3)
V3^i^—K4—K12	169.078 (10)	O22—K16—O1^xv^	83.34 (3)
O1W—K4—K12	72.648 (19)	O2^xiv^—K16—O1^xv^	91.27 (3)
O9—K4—K1^i^	46.78 (2)	O22—K16—O23	127.88 (3)
O6—K4—K1^i^	111.60 (2)	O2^xiv^—K16—O23	136.17 (3)
O7^i^—K4—K1^i^	96.99 (2)	O1^xv^—K16—O23	74.64 (3)
O12—K4—K1^i^	113.00 (2)	O22—K16—O6^xiv^	177.28 (3)
O5^i^—K4—K1^i^	46.08 (2)	O2^xiv^—K16—O6^xiv^	87.80 (3)
O2—K4—K1^i^	149.30 (2)	O1^xv^—K16—O6^xiv^	95.22 (3)
V2—K4—K1^i^	129.926 (10)	O23—K16—O6^xiv^	53.63 (3)
V3^i^—K4—K1^i^	74.317 (8)	O22—K16—O18^xiv^	130.87 (3)
O1W—K4—K1^i^	61.43 (2)	O2^xiv^—K16—O18^xiv^	121.24 (3)
K12—K4—K1^i^	98.686 (9)	O1^xv^—K16—O18^xiv^	127.70 (3)
O9—K4—K2^i^	117.43 (2)	O23—K16—O18^xiv^	53.32 (2)
O6—K4—K2^i^	44.54 (2)	O6^xiv^—K16—O18^xiv^	51.77 (2)
O7^i^—K4—K2^i^	46.32 (2)	O22—K16—O4W	83.06 (3)
O12—K4—K2^i^	123.63 (2)	O2^xiv^—K16—O4W	120.28 (3)
O5^i^—K4—K2^i^	104.15 (3)	O1^xv^—K16—O4W	145.46 (3)
O2—K4—K2^i^	83.504 (19)	O23—K16—O4W	89.22 (3)
V2—K4—K2^i^	110.947 (9)	O6^xiv^—K16—O4W	99.38 (3)
V3^i^—K4—K2^i^	77.022 (8)	O18^xiv^—K16—O4W	49.22 (3)
O1W—K4—K2^i^	69.70 (2)	O22—K16—V4^xiv^	151.55 (2)
K12—K4—K2^i^	99.779 (9)	O2^xiv^—K16—V4^xiv^	118.13 (2)
K1^i^—K4—K2^i^	118.727 (9)	O1^xv^—K16—V4^xiv^	99.60 (2)
O9—K4—H1A	58.9 (4)	O23—K16—V4^xiv^	31.323 (18)
O6—K4—H1A	34.9 (2)	O6^xiv^—K16—V4^xiv^	30.954 (17)
O7^i^—K4—H1A	87.9 (4)	O18^xiv^—K16—V4^xiv^	30.605 (17)
O12—K4—H1A	116.8 (4)	O4W—K16—V4^xiv^	78.71 (2)
O5^i^—K4—H1A	104.3 (3)	O22—K16—O21	54.38 (3)
O2—K4—H1A	133.3 (2)	O2^xiv^—K16—O21	144.30 (2)
V2—K4—H1A	139.1 (3)	O1^xv^—K16—O21	84.36 (2)
V3^i^—K4—H1A	103.3 (4)	O23—K16—O21	76.57 (2)
O1W—K4—H1A	15.8 (2)	O6^xiv^—K16—O21	127.86 (2)
K12—K4—H1A	66.6 (4)	O18^xiv^—K16—O21	88.12 (2)
K1^i^—K4—H1A	77.2 (2)	O4W—K16—O21	61.99 (2)
K2^i^—K4—H1A	58.6 (3)	V4^xiv^—K16—O21	97.509 (17)
O9—K4—H1B	34.6 (2)	O22—K16—V6	26.13 (2)
O6—K4—H1B	60.2 (4)	O2^xiv^—K16—V6	116.06 (2)
O7^i^—K4—H1B	101.9 (3)	O1^xv^—K16—V6	83.55 (2)
O12—K4—H1B	112.0 (3)	O23—K16—V6	103.56 (2)
O5^i^—K4—H1B	89.6 (4)	O6^xiv^—K16—V6	156.112 (19)
O2—K4—H1B	158.2 (4)	O18^xiv^—K16—V6	111.007 (19)
V2—K4—H1B	143.0 (4)	O4W—K16—V6	70.60 (2)
V3^i^—K4—H1B	103.1 (4)	V4^xiv^—K16—V6	125.600 (10)
O1W—K4—H1B	15.5 (2)	O21—K16—V6	28.263 (15)
K12—K4—H1B	66.1 (4)	O22—K16—K2^xvi^	132.81 (2)
K1^i^—K4—H1B	51.8 (4)	O2^xiv^—K16—K2^xvi^	86.60 (2)
K2^i^—K4—H1B	85.0 (2)	O1^xv^—K16—K2^xvi^	49.776 (19)
H1A—K4—H1B	28.0 (4)	O23—K16—K2^xvi^	52.45 (2)
O22^v^—K5—O2^i^	159.26 (3)	O6^xiv^—K16—K2^xvi^	45.555 (19)
O22^v^—K5—O4^i^	97.67 (3)	O18^xiv^—K16—K2^xvi^	89.387 (18)
O2^i^—K5—O4^i^	61.93 (3)	O4W—K16—K2^xvi^	137.53 (2)
O22^v^—K5—O15	91.51 (3)	V4^xiv^—K16—K2^xvi^	59.086 (7)
O2^i^—K5—O15	106.11 (3)	O21—K16—K2^xvi^	115.820 (18)
O4^i^—K5—O15	137.70 (3)	V6—K16—K2^xvi^	129.641 (10)
O22^v^—K5—O13^iii^	85.98 (3)	O22—K16—V2^xiv^	89.15 (2)
O2^i^—K5—O13^iii^	108.25 (3)	O2^xiv^—K16—V2^xiv^	27.046 (18)
O4^i^—K5—O13^iii^	144.47 (3)	O1^xv^—K16—V2^xiv^	118.00 (2)
O15—K5—O13^iii^	77.08 (3)	O23—K16—V2^xiv^	142.88 (2)
O22^v^—K5—O2^iii^	87.38 (3)	O6^xiv^—K16—V2^xiv^	89.499 (19)
O2^i^—K5—O2^iii^	87.47 (3)	O18^xiv^—K16—V2^xiv^	102.737 (19)
O4^i^—K5—O2^iii^	86.13 (3)	O4W—K16—V2^xiv^	93.37 (2)
O15—K5—O2^iii^	135.68 (3)	V4^xiv^—K16—V2^xiv^	113.420 (10)
O13^iii^—K5—O2^iii^	58.63 (3)	O21—K16—V2^xiv^	136.136 (17)
O22^v^—K5—O5W	82.77 (7)	V6—K16—V2^xiv^	112.214 (9)
O2^i^—K5—O5W	90.18 (7)	K2^xvi^—K16—V2^xiv^	106.783 (10)
O4^i^—K5—O5W	62.62 (6)	O22—K16—H4A	99.97 (18)
O15—K5—O5W	77.91 (6)	O2^xiv^—K16—H4A	120.4 (4)
O13^iii^—K5—O5W	152.21 (6)	O1^xv^—K16—H4A	148.0 (4)
O2^iii^—K5—O5W	145.37 (6)	O23—K16—H4A	78.6 (3)
O22^v^—K5—V2^i^	128.57 (2)	O6^xiv^—K16—H4A	82.48 (18)
O2^i^—K5—V2^i^	31.036 (19)	O18^xiv^—K16—H4A	32.8 (2)
O4^i^—K5—V2^i^	30.92 (2)	O4W—K16—H4A	16.91 (18)
O15—K5—V2^i^	125.59 (2)	V4^xiv^—K16—H4A	63.0 (2)
O13^iii^—K5—V2^i^	131.87 (2)	O21—K16—H4A	72.6 (3)
O2^iii^—K5—V2^i^	87.252 (19)	V6—K16—H4A	86.1 (2)
O5W—K5—V2^i^	73.65 (6)	K2^xvi^—K16—H4A	122.1 (2)
O22^v^—K5—K17^v^	142.62 (2)	V2^xiv^—K16—H4A	93.9 (4)
O2^i^—K5—K17^v^	57.18 (2)	O7^v^—K17—O4^xiv^	164.31 (3)
O4^i^—K5—K17^v^	119.02 (2)	O7^v^—K17—O1^xiv^	98.90 (3)
O15—K5—K17^v^	66.84 (2)	O4^xiv^—K17—O1^xiv^	88.18 (3)
O13^iii^—K5—K17^v^	60.39 (2)	O7^v^—K17—O2^xv^	81.26 (3)
O2^iii^—K5—K17^v^	88.155 (19)	O4^xiv^—K17—O2^xv^	84.79 (3)
O5W—K5—K17^v^	119.03 (6)	O1^xiv^—K17—O2^xv^	89.85 (3)
V2^i^—K5—K17^v^	88.209 (9)	O7^v^—K17—O4W^xiv^	110.47 (3)
O22^v^—K5—V2^iii^	95.53 (2)	O4^xiv^—K17—O4W^xiv^	77.72 (3)
O2^i^—K5—V2^iii^	90.19 (2)	O1^xiv^—K17—O4W^xiv^	118.21 (3)
O4^i^—K5—V2^iii^	114.17 (3)	O2^xv^—K17—O4W^xiv^	145.93 (3)
O15—K5—V2^iii^	105.82 (2)	O7^v^—K17—O13^xiv^	118.71 (3)
O13^iii^—K5—V2^iii^	30.61 (2)	O4^xiv^—K17—O13^xiv^	55.29 (3)
O2^iii^—K5—V2^iii^	30.677 (18)	O1^xiv^—K17—O13^xiv^	142.38 (3)
O5W—K5—V2^iii^	175.98 (5)	O2^xv^—K17—O13^xiv^	94.83 (3)
V2^i^—K5—V2^iii^	104.853 (9)	O4W^xiv^—K17—O13^xiv^	51.22 (3)
K17^v^—K5—V2^iii^	64.367 (8)	O7^v^—K17—O11^xiv^	75.60 (3)
O22^v^—K5—K14^v^	52.44 (2)	O4^xiv^—K17—O11^xiv^	119.45 (3)
O2^i^—K5—K14^v^	148.06 (2)	O1^xiv^—K17—O11^xiv^	53.30 (2)
O4^i^—K5—K14^v^	150.01 (2)	O2^xv^—K17—O11^xiv^	131.31 (3)
O15—K5—K14^v^	53.00 (2)	O4W^xiv^—K17—O11^xiv^	82.69 (3)
O13^iii^—K5—K14^v^	48.37 (2)	O13^xiv^—K17—O11^xiv^	133.86 (3)
O2^iii^—K5—K14^v^	93.857 (19)	O7^v^—K17—K5^v^	84.49 (2)
O5W—K5—K14^v^	105.81 (6)	O4^xiv^—K17—K5^v^	81.12 (2)
V2^i^—K5—K14^v^	178.573 (10)	O1^xiv^—K17—K5^v^	139.72 (2)
K17^v^—K5—K14^v^	90.929 (10)	O2^xv^—K17—K5^v^	50.732 (18)
V2^iii^—K5—K14^v^	75.770 (8)	O4W^xiv^—K17—K5^v^	97.33 (2)
O22^v^—K5—K1^i^	51.40 (2)	O13^xiv^—K17—K5^v^	51.269 (19)
O2^i^—K5—K1^i^	109.05 (2)	O11^xiv^—K17—K5^v^	158.618 (19)
O4^i^—K5—K1^i^	47.55 (2)	O7^v^—K17—O15^v^	54.57 (2)
O15—K5—K1^i^	118.97 (2)	O4^xiv^—K17—O15^v^	117.74 (3)
O13^iii^—K5—K1^i^	132.06 (2)	O1^xiv^—K17—O15^v^	153.44 (3)
O2^iii^—K5—K1^i^	94.243 (19)	O2^xv^—K17—O15^v^	87.09 (2)
O5W—K5—K1^i^	54.17 (5)	O4W^xiv^—K17—O15^v^	75.89 (3)
V2^i^—K5—K1^i^	78.109 (8)	O13^xiv^—K17—O15^v^	64.17 (2)
K17^v^—K5—K1^i^	165.962 (11)	O11^xiv^—K17—O15^v^	111.64 (2)
V2^iii^—K5—K1^i^	122.002 (10)	K5^v^—K17—O15^v^	48.530 (16)
K14^v^—K5—K1^i^	102.686 (9)	O7^v^—K17—V1^xiv^	98.03 (2)
O10—K6—O17	97.56 (3)	O4^xiv^—K17—V1^xiv^	94.97 (2)
O10—K6—O1W^i^	106.49 (3)	O1^xiv^—K17—V1^xiv^	29.293 (18)
O17—K6—O1W^i^	133.96 (3)	O2^xv^—K17—V1^xiv^	118.80 (2)
O10—K6—O16	84.90 (3)	O4W^xiv^—K17—V1^xiv^	91.91 (2)
O17—K6—O16	77.59 (3)	O13^xiv^—K17—V1^xiv^	133.93 (2)
O1W^i^—K6—O16	141.96 (3)	O11^xiv^—K17—V1^xiv^	29.120 (16)
O10—K6—O7	130.01 (3)	K5^v^—K17—V1^xiv^	168.920 (11)
O17—K6—O7	100.85 (3)	O15^v^—K17—V1^xiv^	140.759 (18)
O1W^i^—K6—O7	92.99 (3)	O7^v^—K17—V2^xiv^	141.30 (2)
O16—K6—O7	55.05 (2)	O4^xiv^—K17—V2^xiv^	28.09 (2)
O10—K6—O8	148.88 (3)	O1^xiv^—K17—V2^xiv^	116.14 (2)
O17—K6—O8	56.62 (3)	O2^xv^—K17—V2^xiv^	82.835 (19)
O1W^i^—K6—O8	85.29 (3)	O4W^xiv^—K17—V2^xiv^	68.01 (2)
O16—K6—O8	103.65 (3)	O13^xiv^—K17—V2^xiv^	28.811 (18)
O7—K6—O8	76.31 (3)	O11^xiv^—K17—V2^xiv^	138.497 (19)
O10—K6—V3	98.88 (2)	K5^v^—K17—V2^xiv^	58.397 (8)
O17—K6—V3	103.63 (2)	O15^v^—K17—V2^xiv^	89.649 (17)
O1W^i^—K6—V3	110.47 (2)	V1^xiv^—K17—V2^xiv^	120.528 (9)
O16—K6—V3	31.589 (18)	O7^v^—K17—K2^v^	49.09 (2)
O7—K6—V3	31.575 (17)	O4^xiv^—K17—K2^v^	136.79 (2)
O8—K6—V3	103.79 (2)	O1^xiv^—K17—K2^v^	49.869 (19)
O10—K6—V5	117.97 (3)	O2^xv^—K17—K2^v^	85.217 (19)
O17—K6—V5	31.715 (19)	O4W^xiv^—K17—K2^v^	127.12 (2)
O1W^i^—K6—V5	102.69 (2)	O13^xiv^—K17—K2^v^	167.70 (2)
O16—K6—V5	103.35 (2)	O11^xiv^—K17—K2^v^	47.526 (17)
O7—K6—V5	101.016 (19)	K5^v^—K17—K2^v^	122.158 (11)
O8—K6—V5	31.152 (18)	O15^v^—K17—K2^v^	103.576 (17)
V3—K6—V5	119.874 (10)	V1^xiv^—K17—K2^v^	54.807 (7)
O10—K6—O5	80.69 (3)	V2^xiv^—K17—K2^v^	161.688 (11)
O17—K6—O5	130.80 (3)	O21—K18—O3^xv^	152.11 (3)
O1W^i^—K6—O5	92.10 (3)	O21—K18—O23	90.76 (3)
O16—K6—O5	53.23 (3)	O3^xv^—K18—O23	98.99 (3)
O7—K6—O5	52.43 (2)	O21—K18—O1^xv^	97.83 (3)
O8—K6—O5	128.50 (3)	O3^xv^—K18—O1^xv^	59.65 (3)
V3—K6—O5	30.924 (18)	O23—K18—O1^xv^	77.22 (3)
V5—K6—O5	150.66 (2)	O21—K18—O20	79.23 (3)
O10—K6—K11	127.32 (2)	O3^xv^—K18—O20	128.42 (3)
O17—K6—K11	51.35 (2)	O23—K18—O20	76.10 (3)
O1W^i^—K6—K11	125.83 (2)	O1^xv^—K18—O20	153.10 (3)
O16—K6—K11	51.03 (2)	O21—K18—O3W	144.27 (6)
O7—K6—K11	49.510 (19)	O3^xv^—K18—O3W	52.16 (6)
O8—K6—K11	52.627 (19)	O23—K18—O3W	53.52 (6)
V3—K6—K11	60.010 (7)	O1^xv^—K18—O3W	76.97 (6)
V5—K6—K11	59.879 (7)	O20—K18—O3W	89.82 (6)
O5—K6—K11	90.905 (19)	O21—K18—O24	102.46 (3)
O10—K6—K1	56.49 (2)	O3^xv^—K18—O24	93.39 (3)
O17—K6—K1	153.13 (2)	O23—K18—O24	124.61 (3)
O1W^i^—K6—K1	67.26 (2)	O1^xv^—K18—O24	149.44 (3)
O16—K6—K1	91.96 (2)	O20—K18—O24	54.89 (3)
O7—K6—K1	92.840 (19)	O3W—K18—O24	98.47 (6)
O8—K6—K1	150.09 (2)	O21—K18—O1W^v^	77.17 (3)
V3—K6—K1	76.581 (8)	O3^xv^—K18—O1W^v^	87.78 (3)
V5—K6—K1	163.454 (10)	O23—K18—O1W^v^	164.40 (3)
O5—K6—K1	45.689 (18)	O1^xv^—K18—O1W^v^	94.47 (3)
K11—K6—K1	136.589 (10)	O20—K18—O1W^v^	110.63 (3)
O10—K6—K10	59.48 (2)	O3W—K18—O1W^v^	138.01 (6)
O17—K6—K10	49.11 (2)	O24—K18—O1W^v^	68.49 (3)
O1W^i^—K6—K10	113.50 (2)	O21—K18—V1^xv^	124.51 (2)
O16—K6—K10	103.62 (2)	O3^xv^—K18—V1^xv^	29.97 (2)
O7—K6—K10	148.91 (2)	O23—K18—V1^xv^	91.66 (2)
O8—K6—K10	89.401 (19)	O1^xv^—K18—V1^xv^	30.185 (18)
V3—K6—K10	134.907 (10)	O20—K18—V1^xv^	153.94 (2)
V5—K6—K10	58.840 (7)	O3W—K18—V1^xv^	64.70 (5)
O5—K6—K10	136.81 (2)	O24—K18—V1^xv^	120.59 (2)
K11—K6—K10	99.960 (9)	O1W^v^—K18—V1^xv^	87.24 (2)
K1—K6—K10	111.801 (10)	O21—K18—V5^xiii^	101.81 (2)
K7B^ix^—K7A—O3W^ii^	112.4 (5)	O3^xv^—K18—V5^xiii^	103.23 (2)
K7B^ix^—K7A—O2W	105.0 (5)	O23—K18—V5^xiii^	95.17 (2)
O3W^ii^—K7A—O2W	141.48 (10)	O1^xv^—K18—V5^xiii^	159.04 (2)
K7B^ix^—K7A—O2W^ix^	63.1 (5)	O20—K18—V5^xiii^	29.916 (18)
O3W^ii^—K7A—O2W^ix^	173.76 (10)	O3W—K18—V5^xiii^	82.80 (6)
O2W—K7A—O2W^ix^	42.16 (9)	O24—K18—V5^xiii^	29.650 (17)
K7B^ix^—K7A—O11	133.8 (5)	O1W^v^—K18—V5^xiii^	96.94 (2)
O3W^ii^—K7A—O11	87.63 (8)	V1^xv^—K18—V5^xiii^	133.057 (10)
O2W—K7A—O11	58.79 (6)	O21—K18—K15	50.99 (2)
O2W^ix^—K7A—O11	92.88 (6)	O3^xv^—K18—K15	137.16 (3)
K7B^ix^—K7A—O3W^ix^	71.6 (5)	O23—K18—K15	120.40 (2)
O3W^ii^—K7A—O3W^ix^	42.47 (11)	O1^xv^—K18—K15	140.49 (2)
O2W—K7A—O3W^ix^	175.78 (9)	O20—K18—K15	55.60 (2)
O2W^ix^—K7A—O3W^ix^	134.12 (9)	O3W—K18—K15	142.49 (5)
O11—K7A—O3W^ix^	125.35 (8)	O24—K18—K15	51.611 (19)
K7B^ix^—K7A—O23^ix^	50.5 (5)	O1W^v^—K18—K15	58.87 (2)
O3W^ii^—K7A—O23^ix^	91.49 (8)	V1^xv^—K18—K15	146.074 (12)
O2W—K7A—O23^ix^	120.43 (7)	V5^xiii^—K18—K15	60.073 (8)
O2W^ix^—K7A—O23^ix^	88.48 (6)	O21—K18—K13	48.37 (2)
O11—K7A—O23^ix^	175.50 (6)	O3^xv^—K18—K13	151.71 (3)
O3W^ix^—K7A—O23^ix^	55.52 (7)	O23—K18—K13	53.43 (2)
K7B^ix^—K7A—O8^x^	64.0 (5)	O1^xv^—K18—K13	112.46 (2)
O3W^ii^—K7A—O8^x^	81.30 (8)	O20—K18—K13	46.446 (19)
O2W—K7A—O8^x^	107.78 (7)	O3W—K18—K13	100.38 (5)
O2W^ix^—K7A—O8^x^	92.67 (7)	O24—K18—K13	98.092 (19)
O11—K7A—O8^x^	79.67 (4)	O1W^v^—K18—K13	120.49 (2)
O3W^ix^—K7A—O8^x^	73.23 (7)	V1^xv^—K18—K13	139.467 (11)
O23^ix^—K7A—O8^x^	104.56 (5)	V5^xiii^—K18—K13	76.362 (8)
K7B^ix^—K7A—O3	164.9 (5)	K15—K18—K13	67.708 (9)
O3W^ii^—K7A—O3	55.00 (7)	O21—K18—H3A	127.1 (5)
O2W—K7A—O3	89.15 (7)	O3^xv^—K18—H3A	65.6 (9)
O2W^ix^—K7A—O3	130.17 (7)	O23—K18—H3A	36.4 (5)
O11—K7A—O3	58.49 (4)	O1^xv^—K18—H3A	73.2 (13)
O3W^ix^—K7A—O3	94.00 (7)	O20—K18—H3A	87.2 (13)
O23^ix^—K7A—O3	117.56 (5)	O3W—K18—H3A	17.4 (5)
O8^x^—K7A—O3	117.30 (5)	O24—K18—H3A	110.6 (10)
K7B^ix^—K7A—O14^xi^	99.3 (5)	O1W^v^—K18—H3A	153.4 (8)
O3W^ii^—K7A—O14^xi^	101.99 (8)	V1^xv^—K18—H3A	70.1 (12)
O2W—K7A—O14^xi^	80.48 (7)	V5^xiii^—K18—H3A	89.0 (12)
O2W^ix^—K7A—O14^xi^	83.30 (6)	K15—K18—H3A	142.7 (13)
O11—K7A—O14^xi^	117.38 (5)	K13—K18—H3A	86.2 (8)
O3W^ix^—K7A—O14^xi^	97.45 (7)	O21—K18—H3B	160.7 (7)
O23^ix^—K7A—O14^xi^	58.50 (4)	O3^xv^—K18—H3B	37.1 (9)
O8^x^—K7A—O14^xi^	162.56 (6)	O23—K18—H3B	70.1 (6)
O3—K7A—O14^xi^	77.37 (4)	O1^xv^—K18—H3B	76.1 (13)
K7B^ix^—K7A—O20^ix^	21.4 (4)	O20—K18—H3B	97.8 (12)
O3W^ii^—K7A—O20^ix^	123.26 (8)	O3W—K18—H3B	16.9 (6)
O2W—K7A—O20^ix^	89.54 (6)	O24—K18—H3B	90.9 (12)
O2W^ix^—K7A—O20^ix^	50.97 (5)	O1W^v^—K18—H3B	121.1 (6)
O11—K7A—O20^ix^	113.01 (5)	V1^xv^—K18—H3B	56.1 (12)
O3W^ix^—K7A—O20^ix^	87.81 (7)	V5^xiii^—K18—H3B	83.0 (13)
O23^ix^—K7A—O20^ix^	71.10 (4)	K15—K18—H3B	141.4 (13)
O8^x^—K7A—O20^ix^	54.29 (3)	K13—K18—H3B	116.6 (7)
O3—K7A—O20^ix^	170.40 (5)	H3A—K18—H3B	33.8 (8)
O14^xi^—K7A—O20^ix^	111.77 (5)	O10—V1—O11	108.99 (5)
K7B^ix^—K7A—V4^xi^	73.1 (5)	O10—V1—O3	110.24 (5)
O3W^ii^—K7A—V4^xi^	99.77 (8)	O11—V1—O3	109.00 (5)
O2W—K7A—V4^xi^	99.77 (6)	O10—V1—O1	110.58 (5)
O2W^ix^—K7A—V4^xi^	83.19 (6)	O11—V1—O1	108.57 (5)
O11—K7A—V4^xi^	146.68 (5)	O3—V1—O1	109.42 (5)
O3W^ix^—K7A—V4^xi^	76.95 (7)	O12—V2—O4	111.29 (5)
O23^ix^—K7A—V4^xi^	29.31 (2)	O12—V2—O13	109.51 (5)
O8^x^—K7A—V4^xi^	133.43 (5)	O4—V2—O13	107.31 (5)
O3—K7A—V4^xi^	99.54 (4)	O12—V2—O2	109.56 (4)
O14^xi^—K7A—V4^xi^	29.33 (2)	O4—V2—O2	109.62 (5)
O20^ix^—K7A—V4^xi^	90.05 (4)	O13—V2—O2	109.52 (5)
K7B^ix^—K7A—H2A	119.1 (10)	O14—V4—O18	110.50 (5)
O3W^ii^—K7A—H2A	122.5 (5)	O14—V4—O23^xiv^	111.43 (5)
O2W—K7A—H2A	21.0 (3)	O18—V4—O23^xiv^	108.09 (5)
O2W^ix^—K7A—H2A	59.8 (6)	O14—V4—O6	110.84 (5)
O11—K7A—H2A	37.8 (3)	O18—V4—O6	108.07 (5)
O3W^ix^—K7A—H2A	163.1 (3)	O23^xiv^—V4—O6	107.78 (5)
O23^ix^—K7A—H2A	141.2 (4)	O24^xiii^—V5—O8	108.98 (5)
O8^x^—K7A—H2A	99.0 (10)	O24^xiii^—V5—O17	109.83 (5)
O3—K7A—H2A	76.0 (9)	O8—V5—O17	107.97 (5)
O14^xi^—K7A—H2A	93.6 (9)	O24^xiii^—V5—O20^xiii^	108.97 (5)
O20^ix^—K7A—H2A	99.9 (10)	O8—V5—O20^xiii^	111.06 (5)
V4^xi^—K7A—H2A	117.7 (7)	O17—V5—O20^xiii^	110.02 (4)
K7A^ix^—K7B—O2W	109.8 (5)	O22—V6—O19	108.09 (4)
K7A^ix^—K7B—O23	123.4 (5)	O22—V6—O21	111.40 (5)
O2W—K7B—O23	96.48 (7)	O19—V6—O21	111.29 (4)
K7A^ix^—K7B—O3W^xii^	60.5 (5)	O22—V6—O9^v^	106.66 (4)
O2W—K7B—O3W^xii^	169.39 (10)	O19—V6—O9^v^	108.92 (4)
O23—K7B—O3W^xii^	92.88 (8)	O21—V6—O9^v^	110.32 (4)
K7A^ix^—K7B—O3W	101.1 (5)	V1—O1—K1	101.78 (4)
O2W—K7B—O3W	148.27 (10)	V1—O1—K2^x^	89.08 (4)
O23—K7B—O3W	59.00 (7)	K1—O1—K2^x^	162.27 (4)
O3W^xii^—K7B—O3W	42.25 (11)	V1—O1—K17^xiv^	96.03 (4)
K7A^ix^—K7B—O2W^ix^	67.7 (5)	K1—O1—K17^xiv^	86.39 (3)
O2W—K7B—O2W^ix^	42.33 (9)	K2^x^—O1—K17^xiv^	78.48 (2)
O23—K7B—O2W^ix^	124.30 (9)	V1—O1—K18^ii^	92.53 (4)
O3W^xii^—K7B—O2W^ix^	127.40 (9)	K1—O1—K18^ii^	95.48 (3)
O3W—K7B—O2W^ix^	168.50 (10)	K2^x^—O1—K18^ii^	98.02 (3)
K7A^ix^—K7B—O8^xiii^	109.2 (5)	K17^xiv^—O1—K18^ii^	170.68 (4)
O2W—K7B—O8^xiii^	99.24 (8)	V1—O1—K16^ii^	164.84 (5)
O23—K7B—O8^xiii^	114.99 (6)	K1—O1—K16^ii^	92.68 (3)
O3W^xii^—K7B—O8^xiii^	81.20 (8)	K2^x^—O1—K16^ii^	78.08 (2)
O3W—K7B—O8^xiii^	76.90 (8)	K17^xiv^—O1—K16^ii^	89.34 (3)
O2W^ix^—K7B—O8^xiii^	108.55 (8)	K18^ii^—O1—K16^ii^	81.46 (2)
K7A^ix^—K7B—O20	156.0 (5)	V2—O2—K5^i^	94.71 (4)
O2W—K7B—O20	56.37 (6)	V2—O2—K4	89.63 (4)
O23—K7B—O20	79.72 (5)	K5^i^—O2—K4	95.23 (3)
O3W^xii^—K7B—O20	130.77 (9)	V2—O2—K16^xiv^	104.18 (4)
O3W—K7B—O20	96.81 (8)	K5^i^—O2—K16^xiv^	160.76 (4)
O2W^ix^—K7B—O20	94.67 (7)	K4—O2—K16^xiv^	88.61 (3)
O8^xiii^—K7B—O20	59.60 (4)	V2—O2—K5^x^	88.68 (3)
K7A^ix^—K7B—O14^xiv^	73.9 (5)	K5^i^—O2—K5^x^	92.53 (3)
O2W—K7B—O14^xiv^	84.19 (8)	K4—O2—K5^x^	172.17 (4)
O23—K7B—O14^xiv^	59.60 (4)	K16^xiv^—O2—K5^x^	84.39 (3)
O3W^xii^—K7B—O14^xiv^	96.32 (8)	V2—O2—K17^ii^	166.69 (5)
O3W—K7B—O14^xiv^	97.62 (9)	K5^i^—O2—K17^ii^	72.08 (2)
O2W^ix^—K7B—O14^xiv^	77.25 (7)	K4—O2—K17^ii^	93.15 (3)
O8^xiii^—K7B—O14^xiv^	174.07 (7)	K16^xiv^—O2—K17^ii^	88.91 (3)
O20—K7B—O14^xiv^	119.53 (6)	K5^x^—O2—K17^ii^	90.24 (3)
K7A^ix^—K7B—O11^ix^	41.3 (4)	V1—O3—K3^xi^	172.50 (6)
O2W—K7B—O11^ix^	90.01 (7)	V1—O3—K18^ii^	96.64 (4)
O23—K7B—O11^ix^	164.58 (7)	K3^xi^—O3—K18^ii^	85.96 (3)
O3W^xii^—K7B—O11^ix^	79.71 (7)	V1—O3—K10	95.94 (4)
O3W—K7B—O11^ix^	119.03 (8)	K3^xi^—O3—K10	78.52 (3)
O2W^ix^—K7B—O11^ix^	54.38 (6)	K18^ii^—O3—K10	149.43 (5)
O8^xiii^—K7B—O11^ix^	77.49 (5)	V1—O3—K7A	93.77 (5)
O20—K7B—O11^ix^	115.36 (6)	K3^xi^—O3—K7A	90.55 (4)
O14^xiv^—K7B—O11^ix^	107.46 (5)	K18^ii^—O3—K7A	124.23 (5)
K7A^ix^—K7B—O3^ix^	13.5 (4)	K10—O3—K7A	82.47 (4)
O2W—K7B—O3^ix^	121.64 (8)	V1—O3—K7B^ix^	95.10 (5)
O23—K7B—O3^ix^	112.86 (6)	K3^xi^—O3—K7B^ix^	89.32 (4)
O3W^xii^—K7B—O3^ix^	49.33 (7)	K18^ii^—O3—K7B^ix^	123.01 (5)
O3W—K7B—O3^ix^	88.32 (8)	K10—O3—K7B^ix^	83.32 (4)
O2W^ix^—K7B—O3^ix^	80.29 (6)	K7A—O3—K7B^ix^	1.66 (5)
O8^xiii^—K7B—O3^ix^	110.67 (6)	V2—O4—K1	168.58 (7)
O20—K7B—O3^ix^	167.20 (7)	V2—O4—K5^i^	93.64 (4)
O14^xiv^—K7B—O3^ix^	71.06 (4)	K1—O4—K5^i^	81.63 (3)
O11^ix^—K7B—O3^ix^	52.25 (3)	V2—O4—K17^xiv^	101.12 (5)
K7A^ix^—K7B—V4^xiv^	101.0 (5)	K1—O4—K17^xiv^	89.81 (3)
O2W—K7B—V4^xiv^	87.11 (7)	K5^i^—O4—K17^xiv^	97.00 (3)
O23—K7B—V4^xiv^	29.78 (3)	V2—O4—K9	108.43 (4)
O3W^xii^—K7B—V4^xiv^	98.68 (8)	K1—O4—K9	74.74 (3)
O3W—K7B—V4^xiv^	79.68 (8)	K5^i^—O4—K9	155.63 (4)
O2W^ix^—K7B—V4^xiv^	99.52 (7)	K17^xiv^—O4—K9	88.94 (3)
O8^xiii^—K7B—V4^xiv^	144.65 (6)	V3—O5—K1	148.02 (7)
O20—K7B—V4^xiv^	97.83 (5)	V3—O5—K8B	93.84 (13)
O14^xiv^—K7B—V4^xiv^	30.17 (2)	K1—O5—K8B	112.61 (11)
O11^ix^—K7B—V4^xiv^	137.59 (6)	V3—O5—K4^i^	91.44 (4)
O3^ix^—K7B—V4^xiv^	94.62 (5)	K1—O5—K4^i^	86.39 (3)
K7A^ix^—K7B—H2B	130.3 (7)	K8B—O5—K4^i^	127.67 (11)
O2W—K7B—H2B	21.7 (2)	V3—O5—K9	95.61 (4)
O23—K7B—H2B	88.2 (10)	K1—O5—K9	75.58 (3)
O3W^xii^—K7B—H2B	164.9 (5)	K8B—O5—K9	74.70 (10)
O3W—K7B—H2B	128.6 (4)	K4^i^—O5—K9	156.08 (5)
O2W^ix^—K7B—H2B	62.7 (4)	V3—O5—K8A	89.76 (4)
O8^xiii^—K7B—H2B	84.7 (8)	K1—O5—K8A	115.47 (4)
O20—K7B—H2B	34.8 (3)	K8B—O5—K8A	5.02 (12)
O14^xiv^—K7B—H2B	97.2 (9)	K4^i^—O5—K8A	130.84 (5)
O11^ix^—K7B—H2B	102.5 (9)	K9—O5—K8A	72.15 (3)
O3^ix^—K7B—H2B	142.9 (4)	V3—O5—K6	73.53 (4)
V4^xiv^—K7B—H2B	89.7 (10)	K1—O5—K6	74.64 (3)
K7A^ix^—K7B—H3A	112.7 (13)	K8B—O5—K6	150.02 (13)
O2W—K7B—H3A	130.9 (7)	K4^i^—O5—K6	80.63 (3)
O23—K7B—H3A	38.6 (3)	K9—O5—K6	79.53 (3)
O3W^xii^—K7B—H3A	59.6 (7)	K8A—O5—K6	145.48 (4)
O3W—K7B—H3A	20.5 (2)	V4—O6—K2^i^	99.77 (4)
O2W^ix^—K7B—H3A	161.2 (8)	V4—O6—K4	156.45 (6)
O8^xiii^—K7B—H3A	89.3 (11)	K2^i^—O6—K4	90.02 (3)
O20—K7B—H3A	89.3 (13)	V4—O6—K12	89.31 (4)
O14^xiv^—K7B—H3A	84.8 (11)	K2^i^—O6—K12	158.29 (4)
O11^ix^—K7B—H3A	138.8 (5)	K4—O6—K12	74.80 (3)
O3^ix^—K7B—H3A	99.2 (12)	V4—O6—K16^xiv^	77.78 (4)
V4^xiv^—K7B—H3A	61.7 (7)	K2^i^—O6—K16^xiv^	75.30 (3)
H2B—K7B—H3A	115.0 (12)	K4—O6—K16^xiv^	84.17 (3)
K7A^ix^—K7B—H2A^ix^	53.6 (10)	K12—O6—K16^xiv^	87.58 (3)
O2W—K7B—H2A^ix^	59.9 (5)	V3—O7—K17^v^	109.16 (4)
O23—K7B—H2A^ix^	142.7 (6)	V3—O7—K4^i^	94.34 (4)
O3W^xii^—K7B—H2A^ix^	109.5 (5)	K17^v^—O7—K4^i^	101.90 (3)
O3W—K7B—H2A^ix^	151.5 (5)	V3—O7—K2	166.74 (5)
O2W^ix^—K7B—H2A^ix^	20.4 (3)	K17^v^—O7—K2	83.03 (3)
O8^xiii^—K7B—H2A^ix^	98.1 (9)	K4^i^—O7—K2	88.01 (3)
O20—K7B—H2A^ix^	104.7 (10)	V3—O7—K11	94.54 (4)
O14^xiv^—K7B—H2A^ix^	87.8 (9)	K17^v^—O7—K11	95.73 (3)
O11^ix^—K7B—H2A^ix^	34.1 (3)	K4^i^—O7—K11	156.41 (4)
O3^ix^—K7B—H2A^ix^	67.0 (9)	K2—O7—K11	78.62 (3)
V4^xiv^—K7B—H2A^ix^	114.8 (8)	V3—O7—K6	79.72 (3)
H2B—K7B—H2A^ix^	77.8 (9)	K17^v^—O7—K6	167.56 (4)
H3A—K7B—H2A^ix^	165.9 (16)	K4^i^—O7—K6	85.68 (3)
O12—K8A—O19	85.21 (3)	K2—O7—K6	87.45 (3)
O12—K8A—O19^v^	98.75 (4)	K11—O7—K6	74.48 (2)
O19—K8A—O19^v^	89.94 (5)	V5—O8—K2	165.75 (6)
O12—K8A—O5W	96.65 (7)	V5—O8—K7B^xiii^	97.57 (5)
O19—K8A—O5W	147.64 (8)	K2—O8—K7B^xiii^	87.96 (4)
O19^v^—K8A—O5W	121.40 (7)	V5—O8—K3^i^	97.67 (4)
O12—K8A—O15	164.49 (4)	K2—O8—K3^i^	91.39 (3)
O19—K8A—O15	87.48 (3)	K7B^xiii^—O8—K3^i^	117.10 (5)
O19^v^—K8A—O15	94.89 (4)	V5—O8—K7A^iii^	103.67 (5)
O5W—K8A—O15	82.36 (7)	K2—O8—K7A^iii^	81.33 (4)
O12—K8A—O5	109.61 (5)	K7B^xiii^—O8—K7A^iii^	6.81 (3)
O19—K8A—O5	93.06 (3)	K3^i^—O8—K7A^iii^	118.47 (4)
O19^v^—K8A—O5	151.64 (3)	V5—O8—K11	89.36 (4)
O5W—K8A—O5	55.77 (7)	K2—O8—K11	77.83 (3)
O15—K8A—O5	57.13 (3)	K7B^xiii^—O8—K11	86.51 (5)
O12—K8A—O9	73.73 (3)	K3^i^—O8—K11	153.93 (4)
O19—K8A—O9	134.09 (5)	K7A^iii^—O8—K11	83.67 (4)
O19^v^—K8A—O9	55.05 (3)	V5—O8—K6	80.37 (4)
O5W—K8A—O9	76.42 (7)	K2—O8—K6	89.65 (3)
O15—K8A—O9	120.64 (3)	K7B^xiii^—O8—K6	158.49 (5)
O5—K8A—O9	132.19 (5)	K3^i^—O8—K6	84.33 (3)
O12—K8A—V6^v^	98.12 (3)	K7A^iii^—O8—K6	155.45 (4)
O19—K8A—V6^v^	120.68 (6)	K11—O8—K6	72.09 (2)
O19^v^—K8A—V6^v^	30.81 (2)	V6^v^—O9—K4	161.62 (5)
O5W—K8A—V6^v^	91.15 (7)	V6^v^—O9—K1^i^	94.43 (4)
O15—K8A—V6^v^	97.37 (2)	K4—O9—K1^i^	88.42 (3)
O5—K8A—V6^v^	138.10 (4)	V6^v^—O9—K12	95.75 (4)
O9—K8A—V6^v^	30.443 (17)	K4—O9—K12	79.10 (3)
O12—K8A—K9	79.12 (2)	K1^i^—O9—K12	166.39 (4)
O19—K8A—K9	49.22 (2)	V6^v^—O9—K8A	78.35 (4)
O19^v^—K8A—K9	139.13 (5)	K4—O9—K8A	83.30 (3)
O5W—K8A—K9	99.24 (7)	K1^i^—O9—K8A	95.66 (4)
O15—K8A—K9	85.75 (2)	K12—O9—K8A	77.64 (3)
O5—K8A—K9	51.54 (3)	V6^v^—O9—K8B	82.65 (11)
O9—K8A—K9	151.69 (3)	K4—O9—K8B	79.17 (10)
V6^v^—K8A—K9	169.48 (5)	K1^i^—O9—K8B	90.44 (12)
O12—K8A—V3	135.43 (4)	K12—O9—K8B	81.96 (11)
O19—K8A—V3	81.58 (2)	K8A—O9—K8B	6.55 (11)
O19^v^—K8A—V3	123.48 (3)	V1—O10—K9	138.32 (6)
O5W—K8A—V3	74.51 (7)	V1—O10—K6	116.49 (5)
O15—K8A—V3	29.45 (2)	K9—O10—K6	95.69 (3)
O5—K8A—V3	30.08 (2)	V1—O10—K13	95.01 (4)
O9—K8A—V3	140.64 (3)	K9—O10—K13	75.46 (3)
V6^v^—K8A—V3	125.147 (14)	K6—O10—K13	136.96 (4)
K9—K8A—V3	60.170 (13)	V1—O10—K1	88.61 (4)
O12—K8A—K12	45.86 (2)	K9—O10—K1	71.89 (3)
O19—K8A—K12	90.59 (4)	K6—O10—K1	78.33 (3)
O19^v^—K8A—K12	53.19 (3)	K13—O10—K1	134.00 (4)
O5W—K8A—K12	113.84 (6)	V1—O10—K10	81.36 (4)
O15—K8A—K12	148.04 (5)	K9—O10—K10	134.66 (4)
O5—K8A—K12	154.77 (4)	K6—O10—K10	75.91 (3)
O9—K8A—K12	45.83 (2)	K13—O10—K10	81.16 (3)
V6^v^—K8A—K12	56.892 (15)	K1—O10—K10	144.36 (3)
K9—K8A—K12	116.405 (18)	V1—O11—K11^x^	164.32 (5)
V3—K8A—K12	171.65 (4)	V1—O11—K2^x^	89.49 (4)
O12—K8A—K14^v^	146.38 (5)	K11^x^—O11—K2^x^	79.00 (3)
O19—K8A—K14^v^	91.21 (4)	V1—O11—K7A	98.30 (5)
O19^v^—K8A—K14^v^	47.75 (2)	K11^x^—O11—K7A	90.21 (4)
O5W—K8A—K14^v^	103.79 (6)	K2^x^—O11—K7A	79.97 (4)
O15—K8A—K14^v^	47.30 (2)	V1—O11—K13	97.71 (4)
O5—K8A—K14^v^	103.96 (2)	K11^x^—O11—K13	88.93 (3)
O9—K8A—K14^v^	85.37 (2)	K2^x^—O11—K13	155.80 (4)
V6^v^—K8A—K14^v^	55.626 (14)	K7A—O11—K13	121.36 (4)
K9—K8A—K14^v^	122.488 (18)	V1—O11—K7B^ix^	103.05 (5)
V3—K8A—K14^v^	76.508 (10)	K11^x^—O11—K7B^ix^	85.29 (4)
K12—K8A—K14^v^	100.91 (3)	K2^x^—O11—K7B^ix^	78.87 (4)
O5W—K8B—O12	105.0 (2)	K7A—O11—K7B^ix^	4.92 (5)
O5W—K8B—O5	63.07 (19)	K13—O11—K7B^ix^	121.31 (5)
O12—K8B—O5	118.0 (2)	V1—O11—K17^xiv^	81.88 (3)
O5W—K8B—O15	88.93 (18)	K11^x^—O11—K17^xiv^	84.39 (3)
O12—K8B—O15	163.6 (2)	K2^x^—O11—K17^xiv^	71.64 (2)
O5—K8B—O15	60.44 (10)	K7A—O11—K17^xiv^	151.61 (4)
O5W—K8B—O19	156.5 (2)	K13—O11—K17^xiv^	86.47 (3)
O12—K8B—O19	80.42 (14)	K7B^ix^—O11—K17^xiv^	150.10 (5)
O5—K8B—O19	94.04 (11)	V2—O12—K8A	123.41 (5)
O15—K8B—O19	83.38 (13)	V2—O12—K14	98.01 (4)
O5W—K8B—O19^v^	122.09 (16)	K8A—O12—K14	96.31 (4)
O12—K8B—O19^v^	91.11 (18)	V2—O12—K8B	116.81 (16)
O5—K8B—O19^v^	149.11 (17)	K8A—O12—K8B	8.01 (14)
O15—K8B—O19^v^	88.70 (16)	K14—O12—K8B	102.39 (14)
O19—K8B—O19^v^	80.0 (2)	V2—O12—K12	147.10 (5)
O5W—K8B—O5W^i^	25.75 (14)	K8A—O12—K12	89.08 (4)
O12—K8B—O5W^i^	84.88 (17)	K14—O12—K12	81.74 (3)
O5—K8B—O5W^i^	59.13 (17)	K8B—O12—K12	95.09 (14)
O15—K8B—O5W^i^	105.7 (2)	V2—O12—K4	92.84 (4)
O19—K8B—O5W^i^	137.88 (18)	K8A—O12—K4	93.38 (3)
O19^v^—K8B—O5W^i^	139.83 (15)	K14—O12—K4	158.18 (4)
O5W—K8B—O9	80.03 (14)	K8B—O12—K4	89.33 (11)
O12—K8B—O9	72.60 (10)	K12—O12—K4	78.91 (3)
O5—K8B—O9	143.0 (2)	V2—O13—K14^xiv^	148.13 (5)
O15—K8B—O9	119.30 (14)	V2—O13—K5^x^	93.59 (4)
O19—K8B—O9	123.0 (2)	K14^xiv^—O13—K5^x^	78.02 (3)
O19^v^—K8B—O9	52.12 (9)	V2—O13—K14	89.81 (4)
O5W^i^—K8B—O9	88.92 (14)	K14^xiv^—O13—K14	90.73 (3)
O5W—K8B—V3	81.34 (18)	K5^x^—O13—K14	163.70 (4)
O12—K8B—V3	141.42 (16)	V2—O13—K17^xiv^	90.44 (4)
O5—K8B—V3	31.36 (5)	K14^xiv^—O13—K17^xiv^	113.91 (3)
O15—K8B—V3	30.92 (5)	K5^x^—O13—K17^xiv^	68.34 (2)
O19—K8B—V3	80.72 (10)	K14—O13—K17^xiv^	127.63 (3)
O19^v^—K8B—V3	118.16 (16)	V4—O14—K10^vi^	175.32 (5)
O5W^i^—K8B—V3	87.01 (18)	V4—O14—K11^v^	90.80 (4)
O9—K8B—V3	144.91 (13)	K10^vi^—O14—K11^v^	90.10 (3)
O5W—K8B—K9	108.8 (2)	V4—O14—K3	104.42 (4)
O12—K8B—K9	80.11 (10)	K10^vi^—O14—K3	76.00 (2)
O5—K8B—K9	54.03 (8)	K11^v^—O14—K3	158.44 (4)
O15—K8B—K9	87.24 (10)	V4—O14—K7A^vi^	93.11 (5)
O19—K8B—K9	48.88 (6)	K10^vi^—O14—K7A^vi^	82.24 (4)
O19^v^—K8B—K9	128.8 (2)	K11^v^—O14—K7A^vi^	107.43 (4)
O5W^i^—K8B—K9	89.85 (17)	K3—O14—K7A^vi^	87.23 (4)
O9—K8B—K9	152.69 (13)	V4—O14—K7B^xiv^	87.01 (5)
V3—K8B—K9	62.19 (7)	K10^vi^—O14—K7B^xiv^	88.32 (4)
O5W—K8B—V6^v^	93.41 (14)	K11^v^—O14—K7B^xiv^	104.45 (4)
O12—K8B—V6^v^	93.86 (14)	K3—O14—K7B^xiv^	91.72 (4)
O5—K8B—V6^v^	143.61 (16)	K7A^vi^—O14—K7B^xiv^	6.83 (2)
O15—K8B—V6^v^	93.96 (13)	V4—O14—K15^v^	110.47 (4)
O19—K8B—V6^v^	109.2 (2)	K10^vi^—O14—K15^v^	74.19 (2)
O19^v^—K8B—V6^v^	29.25 (5)	K11^v^—O14—K15^v^	74.98 (2)
O5W^i^—K8B—V6^v^	111.05 (14)	K3—O14—K15^v^	85.29 (3)
O9—K8B—V6^v^	29.25 (4)	K7A^vi^—O14—K15^v^	156.36 (4)
V3—K8B—V6^v^	124.12 (12)	K7B^xiv^—O14—K15^v^	162.47 (4)
K9—K8B—V6^v^	157.8 (3)	V3—O15—K5	113.51 (4)
O5W—K8B—V2	92.00 (19)	V3—O15—K12^v^	92.84 (4)
O12—K8B—V2	23.77 (6)	K5—O15—K12^v^	152.88 (4)
O5—K8B—V2	94.41 (16)	V3—O15—K14^v^	171.28 (5)
O15—K8B—V2	150.98 (16)	K5—O15—K14^v^	75.18 (2)
O19—K8B—V2	84.36 (10)	K12^v^—O15—K14^v^	78.61 (2)
O19^v^—K8B—V2	114.86 (15)	V3—O15—K8B	90.11 (13)
O5W^i^—K8B—V2	67.83 (12)	K5—O15—K8B	86.24 (10)
O9—K8B—V2	89.36 (9)	K12^v^—O15—K8B	100.58 (14)
V3—K8B—V2	120.82 (15)	K14^v^—O15—K8B	89.78 (14)
K9—K8B—V2	65.04 (7)	V3—O15—K8A	95.07 (4)
V6^v^—K8B—V2	114.91 (11)	K5—O15—K8A	89.45 (3)
O10—K9—O19	154.38 (3)	K12^v^—O15—K8A	94.66 (4)
O10—K9—O5	89.85 (3)	K14^v^—O15—K8A	84.02 (4)
O19—K9—O5	99.19 (3)	K8B—O15—K8A	7.52 (13)
O10—K9—O21	95.80 (3)	V3—O15—K17^v^	84.01 (3)
O19—K9—O21	61.32 (3)	K5—O15—K17^v^	64.63 (2)
O5—K9—O21	136.38 (3)	K12^v^—O15—K17^v^	114.63 (3)
O10—K9—O16	84.85 (3)	K14^v^—O15—K17^v^	101.03 (3)
O19—K9—O16	79.92 (3)	K8B—O15—K17^v^	144.50 (15)
O5—K9—O16	57.94 (3)	K8A—O15—K17^v^	150.71 (4)
O21—K9—O16	79.49 (3)	V3—O16—K15	174.69 (5)
O10—K9—O4	95.44 (3)	V3—O16—K12^v^	94.55 (4)
O19—K9—O4	109.85 (3)	K15—O16—K12^v^	88.20 (3)
O5—K9—O4	77.71 (3)	V3—O16—K11	93.61 (4)
O21—K9—O4	143.95 (3)	K15—O16—K11	81.52 (3)
O16—K9—O4	135.65 (3)	K12^v^—O16—K11	97.15 (3)
O10—K9—O4W	92.45 (3)	V3—O16—K6	84.63 (4)
O19—K9—O4W	90.45 (3)	K15—O16—K6	92.10 (3)
O5—K9—O4W	152.41 (3)	K12^v^—O16—K6	173.28 (4)
O21—K9—O4W	70.69 (3)	K11—O16—K6	76.27 (2)
O16—K9—O4W	149.65 (3)	V3—O16—K9	92.70 (4)
O4—K9—O4W	74.70 (3)	K15—O16—K9	91.12 (3)
O10—K9—V6	126.50 (3)	K12^v^—O16—K9	102.67 (3)
O19—K9—V6	30.637 (19)	K11—O16—K9	158.63 (4)
O5—K9—V6	123.01 (2)	K6—O16—K9	84.03 (3)
O21—K9—V6	30.933 (18)	V5—O17—K6	91.46 (4)
O16—K9—V6	80.92 (2)	V5—O17—K10^xiii^	94.99 (4)
O4—K9—V6	129.46 (2)	K6—O17—K10^xiii^	170.09 (4)
O4W—K9—V6	76.50 (2)	V5—O17—K10	95.82 (4)
O10—K9—K8B	139.95 (13)	K6—O17—K10	82.99 (3)
O19—K9—K8B	57.70 (14)	K10^xiii^—O17—K10	103.78 (3)
O5—K9—K8B	51.27 (12)	V5—O17—K15	171.47 (5)
O21—K9—K8B	118.28 (13)	K6—O17—K15	93.76 (3)
O16—K9—K8B	81.68 (7)	K10^xiii^—O17—K15	78.99 (2)
O4—K9—K8B	69.86 (10)	K10—O17—K15	91.51 (3)
O4W—K9—K8B	117.26 (8)	V5—O17—K11	94.31 (4)
V6—K9—K8B	88.27 (14)	K6—O17—K11	79.70 (3)
O10—K9—K1	60.56 (2)	K10^xiii^—O17—K11	92.29 (3)
O19—K9—K1	141.60 (2)	K10—O17—K11	160.13 (4)
O5—K9—K1	50.67 (2)	K15—O17—K11	80.01 (2)
O21—K9—K1	156.37 (2)	V4—O18—K11^v^	97.90 (4)
O16—K9—K1	97.17 (2)	V4—O18—K12	97.00 (4)
O4—K9—K1	47.91 (2)	K11^v^—O18—K12	99.82 (3)
O4W—K9—K1	107.78 (2)	V4—O18—K13^xiv^	96.89 (4)
V6—K9—K1	172.158 (11)	K11^v^—O18—K13^xiv^	90.16 (3)
K8B—K9—K1	83.92 (14)	K12—O18—K13^xiv^	161.60 (4)
O10—K9—K13	55.95 (2)	V4—O18—K16^xiv^	77.54 (3)
O19—K9—K13	108.40 (2)	K11^v^—O18—K16^xiv^	168.67 (4)
O5—K9—K13	144.40 (3)	K12—O18—K16^xiv^	91.08 (3)
O21—K9—K13	50.475 (19)	K13^xiv^—O18—K16^xiv^	80.20 (2)
O16—K9—K13	104.72 (2)	V6—O19—K9	97.32 (4)
O4—K9—K13	111.97 (2)	V6—O19—K8A	174.97 (5)
O4W—K9—K13	51.10 (2)	K9—O19—K8A	79.28 (4)
V6—K9—K13	78.503 (9)	V6—O19—K8A^v^	93.40 (4)
K8B—K9—K13	164.02 (12)	K9—O19—K8A^v^	169.24 (4)
K1—K9—K13	109.323 (10)	K8A—O19—K8A^v^	90.06 (5)
O10—K9—K8A	145.70 (3)	V6—O19—K14	92.20 (4)
O19—K9—K8A	51.50 (3)	K9—O19—K14	93.15 (3)
O5—K9—K8A	56.31 (3)	K8A—O19—K14	91.70 (3)
O21—K9—K8A	112.24 (3)	K8A^v^—O19—K14	85.66 (3)
O16—K9—K8A	81.47 (2)	V6—O19—K8B	169.81 (13)
O4—K9—K8A	73.67 (3)	K9—O19—K8B	73.42 (13)
O4W—K9—K8A	114.72 (3)	K8A—O19—K8B	5.89 (11)
V6—K9—K8A	82.09 (3)	K8A^v^—O19—K8B	95.94 (15)
K8B—K9—K8A	6.22 (11)	K14—O19—K8B	92.55 (8)
K1—K9—K8A	90.11 (3)	V6—O19—K12^v^	86.20 (3)
K13—K9—K8A	158.32 (3)	K9—O19—K12^v^	100.83 (3)
O17^xiii^—K10—O17	76.22 (3)	K8A—O19—K12^v^	90.78 (3)
O17^xiii^—K10—O20	61.09 (3)	K8A^v^—O19—K12^v^	80.58 (3)
O17—K10—O20	91.46 (3)	K14—O19—K12^v^	166.02 (4)
O17^xiii^—K10—O14^xi^	83.63 (3)	K8B—O19—K12^v^	91.33 (8)
O17—K10—O14^xi^	135.76 (3)	V6—O19—K8B^v^	89.37 (10)
O20—K10—O14^xi^	112.55 (3)	K9—O19—K8B^v^	173.16 (10)
O17^xiii^—K10—O3	165.49 (3)	K8A—O19—K8B^v^	94.13 (12)
O17—K10—O3	115.52 (3)	K8A^v^—O19—K8B^v^	4.09 (9)
O20—K10—O3	124.13 (3)	K14—O19—K8B^v^	85.15 (7)
O14^xi^—K10—O3	81.92 (3)	K8B—O19—K8B^v^	100.0 (2)
O17^xiii^—K10—O24^xiii^	95.06 (3)	K12^v^—O19—K8B^v^	80.95 (7)
O17—K10—O24^xiii^	59.50 (3)	V5^xiii^—O20—K13	170.44 (5)
O20—K10—O24^xiii^	147.32 (3)	V5^xiii^—O20—K10	93.76 (4)
O14^xi^—K10—O24^xiii^	84.06 (3)	K13—O20—K10	94.96 (3)
O3—K10—O24^xiii^	84.73 (3)	V5^xiii^—O20—K7B	91.59 (5)
O17^xiii^—K10—O2W	92.00 (5)	K13—O20—K7B	87.61 (4)
O17—K10—O2W	142.85 (5)	K10—O20—K7B	118.82 (5)
O20—K10—O2W	52.88 (5)	V5^xiii^—O20—K18	90.84 (4)
O14^xi^—K10—O2W	75.65 (5)	K13—O20—K18	79.61 (3)
O3—K10—O2W	83.21 (5)	K10—O20—K18	157.46 (4)
O24^xiii^—K10—O2W	157.63 (5)	K7B—O20—K18	83.04 (4)
O17^xiii^—K10—O10	138.17 (3)	V5^xiii^—O20—K15	89.04 (4)
O17—K10—O10	83.91 (3)	K13—O20—K15	87.64 (3)
O20—K10—O10	83.38 (3)	K10—O20—K15	86.31 (3)
O14^xi^—K10—O10	133.36 (3)	K7B—O20—K15	154.73 (5)
O3—K10—O10	54.85 (3)	K18—O20—K15	71.70 (2)
O24^xiii^—K10—O10	106.00 (3)	V5^xiii^—O20—K7A^ix^	90.49 (5)
O2W—K10—O10	82.07 (5)	K13—O20—K7A^ix^	89.09 (4)
O17^xiii^—K10—V5^xiii^	30.487 (19)	K10—O20—K7A^ix^	116.57 (4)
O17—K10—V5^xiii^	81.78 (2)	K7B—O20—K7A^ix^	2.60 (5)
O20—K10—V5^xiii^	30.645 (19)	K18—O20—K7A^ix^	85.41 (3)
O14^xi^—K10—V5^xiii^	100.06 (2)	K15—O20—K7A^ix^	157.09 (4)
O3—K10—V5^xiii^	153.22 (2)	V6—O21—K13	147.07 (5)
O24^xiii^—K10—V5^xiii^	122.05 (2)	V6—O21—K18	116.27 (4)
O2W—K10—V5^xiii^	71.63 (4)	K13—O21—K18	82.51 (3)
O10—K10—V5^xiii^	111.043 (19)	V6—O21—K15	116.73 (4)
O17^xiii^—K10—V5	81.35 (2)	K13—O21—K15	92.34 (3)
O17—K10—V5	29.990 (18)	K18—O21—K15	79.22 (2)
O20—K10—V5	118.66 (2)	V6—O21—K9	89.20 (4)
O14^xi^—K10—V5	108.79 (2)	K13—O21—K9	74.70 (2)
O3—K10—V5	104.55 (2)	K18—O21—K9	154.52 (4)
O24^xiii^—K10—V5	29.978 (18)	K15—O21—K9	90.31 (3)
O2W—K10—V5	171.39 (4)	V6—O21—K16	81.54 (3)
O10—K10—V5	99.215 (19)	K13—O21—K16	77.83 (2)
V5^xiii^—K10—V5	100.080 (8)	K18—O21—K16	74.60 (2)
O17^xiii^—K10—V1	157.40 (2)	K15—O21—K16	152.97 (3)
O17—K10—V1	109.80 (2)	K9—O21—K16	110.71 (3)
O20—K10—V1	96.55 (2)	V6—O22—K16	112.64 (4)
O14^xi^—K10—V1	103.89 (2)	V6—O22—K5^v^	149.04 (5)
O3—K10—V1	29.87 (2)	K16—O22—K5^v^	98.28 (3)
O24^xiii^—K10—V1	106.81 (2)	V6—O22—K14	95.01 (4)
O2W—K10—V1	69.90 (4)	K16—O22—K14	97.12 (3)
O10—K10—V1	29.473 (17)	K5^v^—O22—K14	79.21 (3)
V5^xiii^—K10—V1	127.187 (10)	V6—O22—K1^xv^	93.94 (4)
V5—K10—V1	115.109 (9)	K16—O22—K1^xv^	99.74 (3)
O17^xiii^—K10—K3^xi^	120.51 (2)	K5^v^—O22—K1^xv^	81.75 (3)
O17—K10—K3^xi^	105.61 (2)	K14—O22—K1^xv^	156.14 (4)
O20—K10—K3^xi^	162.82 (2)	V4^xiv^—O23—K7B	100.73 (6)
O14^xi^—K10—K3^xi^	53.25 (2)	V4^xiv^—O23—K18	162.65 (5)
O3—K10—K3^xi^	49.93 (2)	K7B—O23—K18	91.64 (5)
O24^xiii^—K10—K3^xi^	48.000 (19)	V4^xiv^—O23—K7A^ix^	96.46 (5)
O2W—K10—K3^xi^	110.62 (4)	K7B—O23—K7A^ix^	6.14 (4)
O10—K10—K3^xi^	100.054 (19)	K18—O23—K7A^ix^	96.73 (4)
V5^xiii^—K10—K3^xi^	148.705 (10)	V4^xiv^—O23—K2^xvi^	89.45 (4)
V5—K10—K3^xi^	77.634 (8)	K7B—O23—K2^xvi^	116.21 (5)
V1—K10—K3^xi^	79.711 (8)	K18—O23—K2^xvi^	95.99 (3)
O17^xiii^—K10—H2B	82.1 (8)	K7A^ix^—O23—K2^xvi^	112.05 (4)
O17—K10—H2B	125.5 (3)	V4^xiv^—O23—K16	84.47 (4)
O20—K10—H2B	35.2 (3)	K7B—O23—K16	167.82 (5)
O14^xi^—K10—H2B	89.2 (6)	K18—O23—K16	81.15 (3)
O3—K10—H2B	96.4 (7)	K7A^ix^—O23—K16	173.30 (5)
O24^xiii^—K10—H2B	172.9 (8)	K2^xvi^—O23—K16	74.56 (2)
O2W—K10—H2B	17.7 (3)	V4^xiv^—O23—K13	91.34 (4)
O10—K10—H2B	80.2 (9)	K7B—O23—K13	87.24 (4)
V5^xiii^—K10—H2B	57.1 (6)	K18—O23—K13	77.03 (3)
V5—K10—H2B	153.9 (3)	K7A^ix^—O23—K13	91.75 (4)
V1—K10—H2B	76.8 (9)	K2^xvi^—O23—K13	155.95 (4)
K3^xi^—K10—H2B	128.4 (3)	K16—O23—K13	81.59 (3)
O18^v^—K11—O11^iii^	89.32 (3)	V5^xiii^—O24—K3^v^	167.07 (5)
O18^v^—K11—O7	97.33 (3)	V5^xiii^—O24—K10^xiii^	93.21 (4)
O11^iii^—K11—O7	83.52 (3)	K3^v^—O24—K10^xiii^	78.74 (3)
O18^v^—K11—O17	133.47 (3)	V5^xiii^—O24—K3^xvii^	94.55 (4)
O11^iii^—K11—O17	132.89 (3)	K3^v^—O24—K3^xvii^	84.21 (3)
O7—K11—O17	104.96 (3)	K10^xiii^—O24—K3^xvii^	134.34 (4)
O18^v^—K11—O16	80.80 (3)	V5^xiii^—O24—K15	94.83 (4)
O11^iii^—K11—O16	138.69 (3)	K3^v^—O24—K15	92.99 (3)
O7—K11—O16	58.52 (3)	K10^xiii^—O24—K15	75.74 (2)
O17—K11—O16	77.16 (3)	K3^xvii^—O24—K15	147.73 (4)
O18^v^—K11—O14^v^	60.33 (3)	V5^xiii^—O24—K18	87.01 (4)
O11^iii^—K11—O14^v^	116.17 (3)	K3^v^—O24—K18	105.21 (3)
O7—K11—O14^v^	148.04 (3)	K10^xiii^—O24—K18	147.85 (4)
O17—K11—O14^v^	80.60 (3)	K3^xvii^—O24—K18	77.53 (2)
O16—K11—O14^v^	93.43 (3)	K15—O24—K18	72.22 (2)
O18^v^—K11—O8	168.70 (3)	K6^i^—O1W—K3	89.00 (3)
O11^iii^—K11—O8	79.39 (3)	K6^i^—O1W—K18^v^	103.03 (3)
O7—K11—O8	81.90 (3)	K3—O1W—K18^v^	99.49 (3)
O17—K11—O8	56.99 (3)	K6^i^—O1W—K15^v^	164.69 (4)
O16—K11—O8	107.97 (3)	K3—O1W—K15^v^	81.87 (3)
O14^v^—K11—O8	124.60 (3)	K18^v^—O1W—K15^v^	66.72 (2)
O18^v^—K11—V4^v^	30.12 (2)	K6^i^—O1W—K4	79.20 (3)
O11^iii^—K11—V4^v^	102.44 (2)	K3—O1W—K4	124.54 (4)
O7—K11—V4^v^	125.29 (2)	K18^v^—O1W—K4	135.96 (4)
O17—K11—V4^v^	108.72 (2)	K15^v^—O1W—K4	116.11 (3)
O16—K11—V4^v^	88.73 (2)	K6^i^—O1W—H1A	104.5 (14)
O14^v^—K11—V4^v^	30.347 (18)	K3—O1W—H1A	72.4 (13)
O8—K11—V4^v^	152.79 (2)	K18^v^—O1W—H1A	151.1 (14)
O18^v^—K11—V3	82.03 (2)	K15^v^—O1W—H1A	84.5 (13)
O11^iii^—K11—V3	109.22 (2)	K4—O1W—H1A	59.2 (13)
O7—K11—V3	30.220 (19)	K6^i^—O1W—H1B	106.9 (13)
O17—K11—V3	97.34 (2)	K3—O1W—H1B	163.7 (14)
O16—K11—V3	29.881 (19)	K18^v^—O1W—H1B	81.1 (13)
O14^v^—K11—V3	118.63 (2)	K15^v^—O1W—H1B	83.4 (13)
O8—K11—V3	101.95 (2)	K4—O1W—H1B	57.0 (13)
V4^v^—K11—V3	102.933 (9)	H1A—O1W—H1B	99.1 (18)
O18^v^—K11—V5	157.35 (2)	K7B—O2W—K7A	144.94 (10)
O11^iii^—K11—V5	102.93 (2)	K7B—O2W—K7A^ix^	7.17 (4)
O7—K11—V5	102.88 (2)	K7A—O2W—K7A^ix^	137.84 (9)
O17—K11—V5	30.046 (18)	K7B—O2W—K7B^ix^	137.67 (9)
O16—K11—V5	101.08 (2)	K7A—O2W—K7B^ix^	7.33 (3)
O14^v^—K11—V5	97.033 (19)	K7A^ix^—O2W—K7B^ix^	130.55 (9)
O8—K11—V5	30.065 (18)	K7B—O2W—K13	88.43 (7)
V4^v^—K11—V5	127.324 (9)	K7A—O2W—K13	123.15 (9)
V3—K11—V5	110.930 (9)	K7A^ix^—O2W—K13	94.47 (7)
O18^v^—K11—K2	125.61 (2)	K7B^ix^—O2W—K13	128.86 (9)
O11^iii^—K11—K2	51.13 (2)	K7B—O2W—K10	119.55 (9)
O7—K11—K2	49.631 (19)	K7A—O2W—K10	82.17 (7)
O17—K11—K2	99.48 (2)	K7A^ix^—O2W—K10	124.07 (9)
O16—K11—K2	104.27 (2)	K7B^ix^—O2W—K10	87.83 (7)
O14^v^—K11—K2	161.91 (2)	K13—O2W—K10	83.56 (6)
O8—K11—K2	46.226 (19)	K7B—O2W—H2A	145 (3)
V4^v^—K11—K2	151.008 (10)	K7A—O2W—H2A	58 (3)
V3—K11—K2	79.404 (8)	K7A^ix^—O2W—H2A	147 (3)
V5—K11—K2	76.162 (8)	K7B^ix^—O2W—H2A	63 (3)
O18^v^—K11—K6	133.11 (2)	K13—O2W—H2A	65 (3)
O11^iii^—K11—K6	120.21 (2)	K10—O2W—H2A	81 (3)
O7—K11—K6	56.01 (2)	K7B—O2W—H2B	69 (3)
O17—K11—K6	48.951 (19)	K7A—O2W—H2B	141 (3)
O16—K11—K6	52.70 (2)	K7A^ix^—O2W—H2B	76 (3)
O14^v^—K11—K6	121.11 (2)	K7B^ix^—O2W—H2B	147 (3)
O8—K11—K6	55.28 (2)	K13—O2W—H2B	52 (3)
V4^v^—K11—K6	136.042 (10)	K10—O2W—H2B	59 (3)
V3—K11—K6	55.263 (7)	H2A—O2W—H2B	107 (4)
V5—K11—K6	55.680 (7)	K7A^xv^—O3W—K7B^xii^	7.10 (4)
K2—K11—K6	69.114 (8)	K7A^xv^—O3W—K7B	144.44 (12)
O12—K12—O16^v^	158.12 (3)	K7B^xii^—O3W—K7B	137.75 (11)
O12—K12—O15^v^	99.45 (3)	K7A^xv^—O3W—K7A^ix^	137.53 (11)
O16^v^—K12—O15^v^	61.69 (3)	K7B^xii^—O3W—K7A^ix^	130.72 (11)
O12—K12—O18	110.04 (3)	K7B—O3W—K7A^ix^	7.28 (3)
O16^v^—K12—O18	81.79 (3)	K7A^xv^—O3W—K2^xvi^	81.17 (8)
O15^v^—K12—O18	87.88 (3)	K7B^xii^—O3W—K2^xvi^	82.14 (9)
O12—K12—O9	81.12 (3)	K7B—O3W—K2^xvi^	113.48 (11)
O16^v^—K12—O9	105.88 (3)	K7A^ix^—O3W—K2^xvi^	113.13 (10)
O15^v^—K12—O9	140.15 (3)	K7A^xv^—O3W—K18	127.97 (11)
O18—K12—O9	129.75 (3)	K7B^xii^—O3W—K18	135.06 (11)
O12—K12—O6	90.42 (3)	K7B—O3W—K18	85.33 (8)
O16^v^—K12—O6	111.39 (3)	K7A^ix^—O3W—K18	92.61 (8)
O15^v^—K12—O6	145.44 (3)	K2^xvi^—O3W—K18	91.93 (8)
O18—K12—O6	57.72 (3)	K7A^xv^—O3W—H3A	140 (4)
O9—K12—O6	73.92 (3)	K7B^xii^—O3W—H3A	142 (4)
O12—K12—O19^v^	91.01 (3)	K7B—O3W—H3A	67 (4)
O16^v^—K12—O19^v^	76.51 (3)	K7A^ix^—O3W—H3A	71 (4)
O15^v^—K12—O19^v^	83.18 (3)	K2^xvi^—O3W—H3A	60 (4)
O18—K12—O19^v^	158.26 (3)	K18—O3W—H3A	54 (4)
O9—K12—O19^v^	57.00 (3)	K7A^xv^—O3W—H3B	83 (4)
O6—K12—O19^v^	129.99 (3)	K7B^xii^—O3W—H3B	89 (4)
O12—K12—V3^v^	129.93 (2)	K7B—O3W—H3B	119 (4)
O16^v^—K12—V3^v^	30.78 (2)	K7A^ix^—O3W—H3B	125 (4)
O15^v^—K12—V3^v^	30.963 (19)	K2^xvi^—O3W—H3B	107 (3)
O18—K12—V3^v^	82.61 (2)	K18—O3W—H3B	50 (4)
O9—K12—V3^v^	128.37 (2)	H3A—O3W—H3B	101 (5)
O6—K12—V3^v^	132.65 (2)	K13—O4W—K17^xiv^	94.25 (4)
O19^v^—K12—V3^v^	79.533 (18)	K13—O4W—K9	71.72 (3)
O12—K12—V6^v^	95.66 (2)	K17^xiv^—O4W—K9	85.51 (3)
O16^v^—K12—V6^v^	82.57 (2)	K13—O4W—K16	80.34 (3)
O15^v^—K12—V6^v^	111.57 (2)	K17^xiv^—O4W—K16	157.73 (4)
O18—K12—V6^v^	144.96 (2)	K9—O4W—K16	112.69 (3)
O9—K12—V6^v^	30.239 (19)	K13—O4W—K14	131.27 (4)
O6—K12—V6^v^	100.16 (2)	K17^xiv^—O4W—K14	121.67 (3)
O19^v^—K12—V6^v^	30.061 (17)	K9—O4W—K14	79.00 (3)
V3^v^—K12—V6^v^	99.135 (9)	K16—O4W—K14	76.15 (3)
O12—K12—V4	109.51 (2)	K13—O4W—H4A	72.8 (14)
O16^v^—K12—V4	89.95 (2)	K17^xiv^—O4W—H4A	87.7 (14)
O15^v^—K12—V4	116.66 (2)	K9—O4W—H4A	143.3 (14)
O18—K12—V4	29.691 (19)	K16—O4W—H4A	70.0 (14)
O9—K12—V4	100.06 (2)	K14—O4W—H4A	133.7 (14)
O6—K12—V4	30.244 (18)	K13—O4W—H4B	161.8 (14)
O19^v^—K12—V4	147.226 (19)	K17^xiv^—O4W—H4B	67.9 (14)
V3^v^—K12—V4	103.848 (9)	K9—O4W—H4B	102.1 (14)
V6^v^—K12—V4	119.615 (9)	K16—O4W—H4B	117.6 (14)
O12—K12—K4	50.70 (2)	K14—O4W—H4B	61.4 (14)
O16^v^—K12—K4	147.79 (2)	H4A—O4W—H4B	108.6 (19)
O15^v^—K12—K4	150.07 (2)	O5W^i^—O5W—K8B	104.1 (3)
O18—K12—K4	99.81 (2)	O5W^i^—O5W—K8A	106.8 (3)
O9—K12—K4	48.845 (19)	K8B—O5W—K8A	3.41 (11)
O6—K12—K4	48.686 (19)	O5W^i^—O5W—K1^i^	122.7 (3)
O19^v^—K12—K4	97.772 (19)	K8B—O5W—K1^i^	101.74 (17)
V3^v^—K12—K4	177.147 (10)	K8A—O5W—K1^i^	98.48 (11)
V6^v^—K12—K4	78.018 (8)	O5W^i^—O5W—K8B^i^	50.2 (3)
V4—K12—K4	77.896 (8)	K8B—O5W—K8B^i^	154.25 (14)
O12—K12—K14	48.72 (2)	K8A—O5W—K8B^i^	156.96 (13)
O16^v^—K12—K14	112.78 (2)	K1^i^—O5W—K8B^i^	94.63 (11)
O15^v^—K12—K14	51.219 (19)	O5W^i^—O5W—K5	162.1 (3)
O18—K12—K14	98.45 (2)	K8B—O5W—K5	85.23 (14)
O9—K12—K14	121.51 (2)	K8A—O5W—K5	83.07 (9)
O6—K12—K14	124.94 (2)	K1^i^—O5W—K5	68.72 (6)
O19^v^—K12—K14	91.343 (18)	K8B^i^—O5W—K5	119.49 (12)
V3^v^—K12—K14	82.171 (8)		

Symmetry codes: (i) −*x*, −*y*+1, −*z*+1; (ii) *x*−1, *y*, *z*; (iii) *x*, *y*+1, *z*; (iv) *x*−1, *y*+1, *z*; (v) −*x*+1, −*y*+1, −*z*+1; (vi) *x*, *y*, *z*+1; (vii) *x*−1, *y*, *z*+1; (viii) −*x*, −*y*+1, −*z*+2; (ix) −*x*+1, −*y*, −*z*; (x) *x*, *y*−1, *z*; (xi) *x*, *y*, *z*−1; (xii) −*x*+2, −*y*, −*z*; (xiii) −*x*+1, −*y*+1, −*z*; (xiv) −*x*+1, −*y*, −*z*+1; (xv) *x*+1, *y*, *z*; (xvi) *x*+1, *y*−1, *z*; (xvii) *x*+1, *y*, *z*−1.

### Tripotassium orthovanadate 0.56-hydrate (K3VO4_0.56H2O) Hydrogen-bond geometry (Å, º)

**Table:**

*D*—H···*A*	*D*—H	H···*A*	*D*···*A*	*D*—H···*A*
O1*W*—H1*A*···O6	0.94 (1)	1.72 (1)	2.6483 (15)	167 (2)
O1*W*—H1*B*···O9	0.94 (1)	1.68 (1)	2.6057 (14)	167 (2)
O2*W*—H2*A*···O11	0.97 (1)	1.72 (1)	2.676 (2)	171 (4)
O2*W*—H2*B*···O20	0.96 (1)	1.67 (1)	2.630 (2)	173 (4)
O3*W*—H3*A*···O23	0.96 (1)	1.70 (2)	2.641 (3)	166 (5)
O3*W*—H3*B*···O3^xv^	0.96 (1)	1.70 (3)	2.559 (3)	148 (5)
O4*W*—H4*B*···O13	0.93 (1)	1.71 (1)	2.6303 (14)	168 (2)
O4*W*—H4*A*···O18^xiv^	0.94 (1)	1.76 (1)	2.6710 (14)	162 (2)
O5*W*···O5			2.725 (3)	
O5*W*···O4^i^			3.089 (3)	

Symmetry codes: (i) −*x*, −*y*+1, −*z*+1; (xiv) −*x*+1, −*y*, −*z*+1; (xv) *x*+1, *y*, *z*.

### Tripotassium orthovanadate tetrahydrate (K3VO4_4H2O) Crystal data

**Table:**

K_3_(VO_4_)·4H_2_O	*D*_x_ = 2.222 Mg m^−^^3^
*M**_r_* = 304.30	Mo *K*α radiation, λ = 0.71073 Å
Orthorhombic, *P**m**n*2_1_	Cell parameters from 3576 reflections
*a* = 12.9607 (13) Å	θ = 3.1–31.3°
*b* = 6.2512 (6) Å	µ = 2.47 mm^−^^1^
*c* = 5.6130 (8) Å	*T* = 100 K
*V* = 454.76 (9) Å^3^	Needle, colorless
*Z* = 2	0.10 × 0.04 × 0.03 mm
*F*(000) = 304	

### Tripotassium orthovanadate tetrahydrate (K3VO4_4H2O) Data collection

**Table:**

Stoe STADIVARI diffractometer	1603 independent reflections
Radiation source: Axo_Mo	1018 reflections with *I* > 2s(*I*)
Graded multilayer mirror monochromator	*R*_int_ = 0.057
Detector resolution: 13.33 pixels mm^-1^	θ_max_ = 32.5°, θ_min_ = 3.1°
rotation method, ω scans	*h* = −19→13
Absorption correction: multi-scan (*LANA*; Koziskova *et al.*, 2016)	*k* = −7→9
*T*_min_ = 0.576, *T*_max_ = 0.895	*l* = −8→8
6297 measured reflections	

### Tripotassium orthovanadate tetrahydrate (K3VO4_4H2O) Refinement

**Table:**

Refinement on *F*^2^	Hydrogen site location: difference Fourier map
Least-squares matrix: full	Only H-atom coordinates refined
*R*[*F*^2^ > 2σ(*F*^2^)] = 0.031	*w* = 1/[σ^2^(*F*_o_^2^) + (0.0139*P*)^2^] where *P* = (*F*_o_^2^ + 2*F*_c_^2^)/3
*wR*(*F*^2^) = 0.051	(Δ/σ)_max_ < 0.001
*S* = 0.80	Δρ_max_ = 0.45 e Å^−^^3^
1603 reflections	Δρ_min_ = −0.78 e Å^−^^3^
74 parameters	Absolute structure: Classical Flack method preferred over Parsons because s.u. lower
7 restraints	Absolute structure parameter: 0.05 (5)

### Tripotassium orthovanadate tetrahydrate (K3VO4_4H2O) Special details

**Table:**

Geometry. All esds (except the esd in the dihedral angle between two l.s. planes) are estimated using the full covariance matrix. The cell esds are taken into account individually in the estimation of esds in distances, angles and torsion angles; correlations between esds in cell parameters are only used when they are defined by crystal symmetry. An approximate (isotropic) treatment of cell esds is used for estimating esds involving l.s. planes.

### Tripotassium orthovanadate tetrahydrate (K3VO4_4H2O) Fractional atomic coordinates and isotropic or equivalent isotropic displacement parameters (Å^2^)

**Table:**

	*x*	*y*	*z*	*U*_iso_*/*U*_eq_	
K1	0.33732 (6)	0.61432 (16)	0.1757 (2)	0.0083 (2)	
K2	0.000000	0.8392 (2)	0.6765 (3)	0.0088 (3)	
V1	0.000000	0.20978 (18)	0.12302 (16)	0.0061 (3)	
O1	0.000000	0.2228 (7)	0.4197 (7)	0.0080 (11)	
O2	0.000000	0.4648 (7)	0.0003 (7)	0.0078 (11)	
O3	0.1088 (2)	0.0759 (5)	0.0241 (5)	0.0080 (8)	
O1W	0.1273 (2)	0.6943 (6)	0.3002 (5)	0.0080 (7)	
H1A	0.088 (2)	0.614 (5)	0.213 (7)	0.012*	
H1B	0.119 (3)	0.825 (4)	0.238 (7)	0.012*	
O2W	0.27168 (19)	0.2135 (6)	0.2991 (6)	0.0113 (8)	
H2A	0.215 (2)	0.180 (7)	0.227 (7)	0.017*	
H2B	0.304 (3)	0.117 (6)	0.372 (7)	0.017*	

### Tripotassium orthovanadate tetrahydrate (K3VO4_4H2O) Atomic displacement parameters (Å^2^)

**Table:**

	*U*^11^	*U*^22^	*U*^33^	*U*^12^	*U*^13^	*U*^23^
K1	0.0081 (4)	0.0079 (5)	0.0091 (5)	−0.0005 (4)	−0.0006 (5)	0.0000 (5)
K2	0.0112 (6)	0.0088 (8)	0.0066 (7)	0.000	0.000	−0.0003 (7)
V1	0.0071 (5)	0.0039 (6)	0.0072 (7)	0.000	0.000	−0.0012 (6)
O1	0.011 (2)	0.008 (3)	0.005 (2)	0.000	0.000	−0.004 (2)
O2	0.005 (2)	0.006 (3)	0.013 (2)	0.000	0.000	−0.002 (2)
O3	0.0064 (14)	0.007 (2)	0.0101 (16)	0.0012 (14)	0.0003 (12)	−0.0005 (14)
O1W	0.0118 (15)	0.0039 (19)	0.0083 (18)	−0.0025 (15)	−0.0031 (14)	−0.0024 (15)
O2W	0.0091 (15)	0.009 (2)	0.0162 (19)	0.0035 (15)	−0.0025 (14)	0.0023 (17)

### Tripotassium orthovanadate tetrahydrate (K3VO4_4H2O) Geometric parameters (Å, º)

**Table:**

K1—O2W	2.735 (4)	K2—O1W^vi^	2.829 (3)
K1—O1^i^	2.747 (3)	K2—O2^vii^	2.964 (5)
K1—O2W^i^	2.761 (4)	K2—O2W^ii^	3.056 (3)
K1—O2^ii^	2.830 (3)	K2—O2W^viii^	3.056 (3)
K1—O3^ii^	2.839 (3)	K2—V1^iv^	3.4126 (19)
K1—O1W	2.855 (3)	K2—V1^iii^	3.8753 (19)
K1—O1W^i^	2.894 (3)	K2—H1B	2.90 (4)
K1—V1^ii^	3.4579 (13)	V1—O1	1.667 (4)
K1—K2^i^	3.5333 (15)	V1—O3	1.732 (3)
K1—K1^i^	3.8785 (12)	V1—O3^vi^	1.732 (3)
K1—K1^ii^	3.8785 (12)	V1—O2	1.736 (5)
K1—V1^i^	3.9090 (14)	O1W—H1A	0.87 (2)
K2—O1^iii^	2.798 (5)	O1W—H1B	0.90 (2)
K2—O3^iv^	2.826 (3)	O2W—H2A	0.86 (2)
K2—O3^v^	2.826 (3)	O2W—H2B	0.84 (2)
K2—O1W	2.829 (3)		
			
O2W—K1—O1^i^	135.29 (11)	O3^iv^—K2—K1^ii^	97.07 (7)
O2W—K1—O2W^i^	113.06 (9)	O3^v^—K2—K1^ii^	135.96 (8)
O1^i^—K1—O2W^i^	81.24 (9)	O1W—K2—K1^ii^	52.71 (7)
O2W—K1—O2^ii^	84.77 (11)	O1W^vi^—K2—K1^ii^	95.17 (8)
O1^i^—K1—O2^ii^	80.20 (9)	O2^vii^—K2—K1^ii^	50.72 (6)
O2W^i^—K1—O2^ii^	160.42 (10)	O2W^ii^—K2—K1^ii^	48.38 (7)
O2W—K1—O3^ii^	121.80 (11)	O2W^viii^—K2—K1^ii^	119.44 (8)
O1^i^—K1—O3^ii^	85.35 (10)	V1^iv^—K2—K1^ii^	123.06 (4)
O2W^i^—K1—O3^ii^	112.78 (10)	K1^viii^—K2—K1^ii^	73.27 (4)
O2^ii^—K1—O3^ii^	59.52 (12)	O1^iii^—K2—V1^iii^	22.28 (9)
O2W—K1—O1W	78.57 (9)	O3^iv^—K2—V1^iii^	103.92 (8)
O1^i^—K1—O1W	142.65 (11)	O3^v^—K2—V1^iii^	103.92 (8)
O2W^i^—K1—O1W	68.35 (9)	O1W—K2—V1^iii^	65.97 (7)
O2^ii^—K1—O1W	125.70 (10)	O1W^vi^—K2—V1^iii^	65.98 (7)
O3^ii^—K1—O1W	86.93 (9)	O2^vii^—K2—V1^iii^	164.53 (10)
O2W—K1—O1W^i^	67.86 (10)	O2W^ii^—K2—V1^iii^	104.19 (7)
O1^i^—K1—O1W^i^	75.19 (11)	O2W^viii^—K2—V1^iii^	104.19 (7)
O2W^i^—K1—O1W^i^	77.48 (9)	V1^iv^—K2—V1^iii^	100.55 (5)
O2^ii^—K1—O1W^i^	103.55 (11)	K1^viii^—K2—V1^iii^	118.60 (4)
O3^ii^—K1—O1W^i^	156.55 (9)	K1^ii^—K2—V1^iii^	118.60 (4)
O1W—K1—O1W^i^	116.49 (8)	O1^iii^—K2—H1B	65.7 (5)
O2W—K1—V1^ii^	107.29 (8)	O3^iv^—K2—H1B	109.6 (8)
O1^i^—K1—V1^ii^	78.12 (7)	O3^v^—K2—H1B	149.9 (5)
O2W^i^—K1—V1^ii^	138.07 (8)	O1W—K2—H1B	18.0 (5)
O2^ii^—K1—V1^ii^	29.99 (10)	O1W^vi^—K2—H1B	70.6 (8)
O3^ii^—K1—V1^ii^	29.91 (6)	O2^vii^—K2—H1B	119.7 (5)
O1W—K1—V1^ii^	110.37 (7)	O2W^ii^—K2—H1B	71.0 (8)
O1W^i^—K1—V1^ii^	130.10 (6)	O2W^viii^—K2—H1B	134.5 (8)
O2W—K1—K2^i^	56.65 (6)	V1^iv^—K2—H1B	130.0 (6)
O1^i^—K1—K2^i^	80.80 (9)	K1^viii^—K2—H1B	107.0 (6)
O2W^i^—K1—K2^i^	128.25 (9)	K1^ii^—K2—H1B	70.0 (5)
O2^ii^—K1—K2^i^	54.16 (10)	V1^iii^—K2—H1B	48.6 (5)
O3^ii^—K1—K2^i^	113.57 (7)	O1—V1—O3	110.12 (14)
O1W—K1—K2^i^	135.18 (8)	O1—V1—O3^vi^	110.12 (14)
O1W^i^—K1—K2^i^	51.05 (7)	O3—V1—O3^vi^	109.1 (2)
V1^ii^—K1—K2^i^	83.71 (3)	O1—V1—O2	110.6 (2)
O2W—K1—K1^i^	70.37 (8)	O3—V1—O2	108.45 (14)
O1^i^—K1—K1^i^	101.88 (8)	O3^vi^—V1—O2	108.45 (14)
O2W^i^—K1—K1^i^	44.84 (7)	O1—V1—K2^ix^	140.06 (17)
O2^ii^—K1—K1^i^	146.75 (10)	O3—V1—K2^ix^	55.70 (10)
O3^ii^—K1—K1^i^	153.27 (7)	O3^vi^—V1—K2^ix^	55.70 (10)
O1W—K1—K1^i^	71.64 (7)	O2—V1—K2^ix^	109.37 (15)
O1W^i^—K1—K1^i^	47.14 (6)	O1—V1—K1^x^	135.20 (8)
V1^ii^—K1—K1^i^	176.74 (5)	O3—V1—K1^x^	114.68 (11)
K2^i^—K1—K1^i^	93.06 (4)	O3^vi^—V1—K1^x^	54.85 (11)
O2W—K1—K1^ii^	45.39 (8)	O2—V1—K1^x^	54.55 (9)
O1^i^—K1—K1^ii^	164.37 (7)	K2^ix^—V1—K1^x^	71.50 (3)
O2W^i^—K1—K1^ii^	113.52 (7)	O1—V1—K1^i^	135.20 (8)
O2^ii^—K1—K1^ii^	84.53 (7)	O3—V1—K1^i^	54.85 (11)
O3^ii^—K1—K1^ii^	84.06 (6)	O3^vi^—V1—K1^i^	114.68 (11)
O1W—K1—K1^ii^	48.00 (7)	O2—V1—K1^i^	54.55 (9)
O1W^i^—K1—K1^ii^	111.95 (8)	K2^ix^—V1—K1^i^	71.50 (3)
V1^ii^—K1—K1^ii^	87.00 (2)	K1^x^—V1—K1^i^	75.14 (4)
K2^i^—K1—K1^ii^	92.96 (4)	O1—V1—K2^xi^	39.51 (16)
K1^i^—K1—K1^ii^	92.70 (4)	O3—V1—K2^xi^	88.18 (11)
O2W—K1—V1^i^	128.79 (8)	O3^vi^—V1—K2^xi^	88.18 (11)
O1^i^—K1—V1^i^	21.03 (7)	O2—V1—K2^xi^	150.08 (15)
O2W^i^—K1—V1^i^	63.81 (7)	K2^ix^—V1—K2^xi^	100.55 (5)
O2^ii^—K1—V1^i^	99.10 (7)	K1^x^—V1—K2^xi^	140.55 (2)
O3^ii^—K1—V1^i^	102.83 (7)	K1^i^—V1—K2^xi^	140.55 (2)
O1W—K1—V1^i^	131.25 (7)	O1—V1—K1^ii^	36.25 (7)
O1W^i^—K1—V1^i^	61.57 (7)	O3—V1—K1^ii^	87.20 (10)
V1^ii^—K1—V1^i^	99.09 (3)	O3^vi^—V1—K1^ii^	146.08 (11)
K2^i^—K1—V1^i^	84.54 (3)	O2—V1—K1^ii^	93.25 (12)
K1^i^—K1—V1^i^	81.033 (19)	K2^ix^—V1—K1^ii^	140.70 (3)
K1^ii^—K1—V1^i^	173.10 (4)	K1^x^—V1—K1^ii^	144.65 (4)
O1^iii^—K2—O3^iv^	84.67 (10)	K1^i^—V1—K1^ii^	99.09 (3)
O1^iii^—K2—O3^v^	84.67 (10)	K2^xi^—V1—K1^ii^	62.08 (3)
O3^iv^—K2—O3^v^	59.89 (12)	O1—V1—K1^viii^	36.25 (7)
O1^iii^—K2—O1W	83.67 (10)	O3—V1—K1^viii^	146.08 (11)
O3^iv^—K2—O1W	113.08 (9)	O3^vi^—V1—K1^viii^	87.20 (10)
O3^v^—K2—O1W	166.97 (12)	O2—V1—K1^viii^	93.25 (12)
O1^iii^—K2—O1W^vi^	83.67 (10)	K2^ix^—V1—K1^viii^	140.70 (3)
O3^iv^—K2—O1W^vi^	166.97 (12)	K1^x^—V1—K1^viii^	99.09 (3)
O3^v^—K2—O1W^vi^	113.08 (8)	K1^i^—V1—K1^viii^	144.65 (4)
O1W—K2—O1W^vi^	71.34 (12)	K2^xi^—V1—K1^viii^	62.08 (3)
O1^iii^—K2—O2^vii^	173.20 (14)	K1^ii^—V1—K1^viii^	65.28 (3)
O3^iv^—K2—O2^vii^	89.44 (11)	V1—O1—K1^ii^	122.73 (11)
O3^v^—K2—O2^vii^	89.44 (11)	V1—O1—K1^viii^	122.73 (11)
O1W—K2—O2^vii^	101.82 (10)	K1^ii^—O1—K1^viii^	100.25 (14)
O1W^vi^—K2—O2^vii^	101.82 (11)	V1—O1—K2^xi^	118.2 (2)
O1^iii^—K2—O2W^ii^	102.04 (7)	K1^ii^—O1—K2^xi^	92.75 (10)
O3^iv^—K2—O2W^ii^	54.39 (9)	K1^viii^—O1—K2^xi^	92.75 (10)
O3^v^—K2—O2W^ii^	112.61 (9)	V1—O2—K1^x^	95.45 (14)
O1W—K2—O2W^ii^	64.46 (9)	V1—O2—K1^i^	95.45 (14)
O1W^vi^—K2—O2W^ii^	134.28 (10)	K1^x^—O2—K1^i^	96.33 (13)
O2^vii^—K2—O2W^ii^	77.09 (7)	V1—O2—K2^xii^	165.6 (2)
O1^iii^—K2—O2W^viii^	102.04 (7)	K1^x^—O2—K2^xii^	75.12 (10)
O3^iv^—K2—O2W^viii^	112.61 (9)	K1^i^—O2—K2^xii^	75.12 (10)
O3^v^—K2—O2W^viii^	54.39 (9)	V1—O3—K2^ix^	93.89 (12)
O1W—K2—O2W^viii^	134.28 (10)	V1—O3—K1^i^	95.24 (14)
O1W^vi^—K2—O2W^viii^	64.46 (9)	K2^ix^—O3—K1^i^	90.25 (9)
O2^vii^—K2—O2W^viii^	77.09 (7)	K2—O1W—K1	142.65 (12)
O2W^ii^—K2—O2W^viii^	151.08 (14)	K2—O1W—K1^ii^	76.24 (8)
O1^iii^—K2—V1^iv^	78.27 (9)	K1—O1W—K1^ii^	84.86 (8)
O3^iv^—K2—V1^iv^	30.41 (6)	K2—O1W—H1A	105 (3)
O3^v^—K2—V1^iv^	30.41 (6)	K1—O1W—H1A	109 (3)
O1W—K2—V1^iv^	139.96 (7)	K1^ii^—O1W—H1A	97 (3)
O1W^vi^—K2—V1^iv^	139.96 (7)	K2—O1W—H1B	86 (3)
O2^vii^—K2—V1^iv^	94.92 (10)	K1—O1W—H1B	101 (3)
O2W^ii^—K2—V1^iv^	84.72 (7)	K1^ii^—O1W—H1B	156 (3)
O2W^viii^—K2—V1^iv^	84.72 (7)	H1A—O1W—H1B	104 (3)
O1^iii^—K2—K1^viii^	133.40 (6)	K1—O2W—K1^ii^	89.77 (11)
O3^iv^—K2—K1^viii^	135.96 (8)	K1—O2W—K2^i^	74.97 (7)
O3^v^—K2—K1^viii^	97.07 (7)	K1^ii^—O2W—K2^i^	135.23 (12)
O1W—K2—K1^viii^	95.17 (8)	K1—O2W—H2A	112 (3)
O1W^vi^—K2—K1^viii^	52.71 (7)	K1^ii^—O2W—H2A	91 (3)
O2^vii^—K2—K1^viii^	50.72 (6)	K2^i^—O2W—H2A	134 (3)
O2W^ii^—K2—K1^viii^	119.44 (8)	K1—O2W—H2B	129 (3)
O2W^viii^—K2—K1^viii^	48.38 (7)	K1^ii^—O2W—H2B	99 (3)
V1^iv^—K2—K1^viii^	123.06 (4)	K2^i^—O2W—H2B	63 (3)
O1^iii^—K2—K1^ii^	133.40 (6)	H2A—O2W—H2B	118 (4)

Symmetry codes: (i) −*x*+1/2, −*y*+1, *z*−1/2; (ii) −*x*+1/2, −*y*+1, *z*+1/2; (iii) *x*, *y*+1, *z*; (iv) *x*, *y*+1, *z*+1; (v) −*x*, *y*+1, *z*+1; (vi) −*x*, *y*, *z*; (vii) *x*, *y*, *z*+1; (viii) *x*−1/2, −*y*+1, *z*+1/2; (ix) *x*, *y*−1, *z*−1; (x) *x*−1/2, −*y*+1, *z*−1/2; (xi) *x*, *y*−1, *z*; (xii) *x*, *y*, *z*−1.

### Tripotassium orthovanadate tetrahydrate (K3VO4_4H2O) Hydrogen-bond geometry (Å, º)

**Table:**

*D*—H···*A*	*D*—H	H···*A*	*D*···*A*	*D*—H···*A*
O1*W*—H1*A*···O2	0.87 (3)	1.90 (3)	2.759 (5)	174 (4)
O1*W*—H1*B*···O3^iii^	0.90 (3)	1.98 (3)	2.855 (5)	165 (3)
O2*W*—H2*A*···O3	0.86 (3)	1.90 (3)	2.753 (4)	168 (4)
O2*W*—H2*B*···O3^xiii^	0.84 (4)	1.86 (4)	2.696 (4)	172 (4)

Symmetry codes: (iii) *x*, *y*+1, *z*; (xiii) −*x*+1/2, −*y*, *z*+1/2.
